# Dysregulation of kynurenine metabolism is related to proinflammatory cytokines, attention, and prefrontal cortex volume in schizophrenia

**DOI:** 10.1038/s41380-019-0401-9

**Published:** 2019-04-03

**Authors:** Jochen Kindler, Chai K. Lim, Cynthia Shannon Weickert, Danny Boerrigter, Cherrie Galletly, Dennis Liu, Kelly R. Jacobs, Ryan Balzan, Jason Bruggemann, Maryanne O’Donnell, Rhoshel Lenroot, Gilles J. Guillemin, Thomas W. Weickert

**Affiliations:** 1 School of Psychiatry, University of New South Wales, Randwick, NSW 2031 Australia; 2 Neuroscience Research Australia, Randwick, NSW 2031 Australia; 3 University Hospital of Child and Adolescent Psychiatry and Psychotherapy, University of Bern, 3000 Bern, Switzerland; 4 Department of Biomedical Sciences, Faculty of Medicine and Health Sciences, Macquarie University, Sydney, NSW 2109 Australia; 5 Schizophrenia Research Institute, Randwick, NSW 2010 Australia; 6 Discipline of Psychiatry, School of Medicine, University of Adelaide, Adelaide, SA Australia; 7 Ramsay Health Care (SA) Mental Health, Adelaide, SA Australia; 8 Northern Adelaide Local Health Network, Adelaide, SA Australia; 9 College of Education, Psychology, and Social Work, Flinders University, Adelaide, SA Australia; 10 Kiloh Centre, Prince of Wales Hospital, Randwick, NSW Australia

**Keywords:** Schizophrenia, Molecular biology

## Abstract

The kynurenine pathway (KP) of tryptophan (TRP) catabolism links immune system activation with neurotransmitter signaling. The KP metabolite kynurenic acid (KYNA) is increased in the brains of people with schizophrenia. We tested the extent to which: (1) brain KP enzyme mRNAs, (2) brain KP metabolites, and (3) plasma KP metabolites differed on the basis of elevated cytokines in schizophrenia vs. control groups and the extent to which plasma KP metabolites were associated with cognition and brain volume in patients displaying elevated peripheral cytokines. KP enzyme mRNAs and metabolites were assayed in two independent postmortem brain samples from a total of 71 patients with schizophrenia and 72 controls. Plasma KP metabolites, cognition, and brain volumes were measured in an independent cohort of 96 patients with schizophrenia and 81 healthy controls. Groups were stratified based on elevated vs. normal proinflammatory cytokine mRNA levels. In the prefrontal cortex (PFC), kynurenine (KYN)/TRP ratio, KYNA levels, and mRNA for enzymes, tryptophan dioxygenase (TDO) and kynurenine aminotransferases (KATI/II), were significantly increased in the high cytokine schizophrenia subgroup. KAT mRNAs significantly correlated with mRNA for glial fibrillary acidic protein in patients. In plasma, the high cytokine schizophrenia subgroup displayed an elevated KYN/TRP ratio, which correlated inversely with attention and dorsolateral prefrontal cortex (DLPFC) volume. This study provides further evidence for the role of inflammation in a subgroup of patients with schizophrenia and suggests a molecular mechanism through which inflammation could lead to schizophrenia. Proinflammatory cytokines may elicit conversion of TRP to KYN in the periphery and increase the *N*-methyl-d-aspartate receptor antagonist KYNA via increased KAT mRNA and possibly more enzyme synthesis activity in brain astrocytes,  leading to DLPFC volume loss, and attention impairment in schizophrenia.

## Introduction

In addition to disturbances in neurotransmitter systems, recent genetic [1], molecular neuropathology [2], and clinical studies [3] have highlighted the importance of immunological factors in the pathogenesis and treatment of schizophrenia. Low-grade inflammation is found in both postmortem brain tissue [2, 4] and in plasma of approximately 40% of people with schizophrenia (termed a “high-cytokine biotype”) [5]. While considerable evidence suggests a role for inflammation in the pathophysiology of schizophrenia, the mechanism by which neuroinflammation influences neurotransmitter systems is unknown. However, neuroactive degradation products of tryptophan (TRP) are produced via the kynurenine pathway (KP), which is regulated by the immune system. The breakdown of TRP via the KP is activated by proinflammatory cytokines [6–8] inducing a catabolic cascade that produces metabolites that can either block or activate neurotransmitter receptors in the brain [9]. Therefore, the KP is considered to link the immune and neurotransmitter systems.

**Fig. 1:**
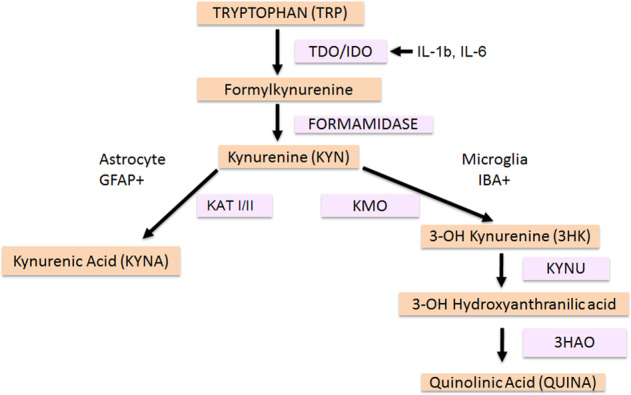
Overview of the kynurenine pathway (KP). TDO tryptophan-2,3-dioxygenase, IDO indoleamine 2,3 dioxygenase, IL-1β interleukin 1 beta, IL-6 interleukin 6, KMO kynurenine 3 monooxygenase, KAT kynurenine amino transferase, GFAP glial fibrillary acidic protein, IBA ionized calcium-binding adaptor molecule, KYNU kynureninase, 3HAO 3-hydroxyanthranilic acid 3,4-dioxygenase

While TRP is commonly thought of as a precursor of the neurotransmitter serotonin, TRP can also be degraded to kynurenine (KYN) [10, 11]. KYN is catabolized to either kynurenic acid (KYNA) via kynurenine aminotransferases (KATs [12], mostly located in brain astrocytes) or to 3-hydroxykynurenine (3-HK) via kynurenine 3-monooxygenase (KMO [13], mostly located in brain microglia) and eventually to quinolinic acid (QUINA). KATI and KATII are the biosynthetic enzymes of KYNA in the mammalian brain [6]. For an overview of the KP, see Fig. 1. Proinflammatory cytokines, e.g., interleukin (IL)-1β, interferon-γ, and IL-6, stimulate an important pathway in macrophages featuring indoleamine 2,3 dioxygenase (IDO) [6, 7], the rate-limiting enzyme degrading TRP into KYN, and KYN can cross the blood–brain barrier (BBB). KYNA, 3-HK, and QUINA possess neuroactive properties and are known to modulate dopaminergic, nicotinergic, and glutamatergic neurotransmission [14–16]. Whereas QUINA is an *N*-methyl-d-aspartate receptor (NMDAR) agonist that can cause excitotoxic neurodegeneration and 3-HK can cause neuronal apoptosis, KYNA is an NMDAR antagonist that is considered to protect against excitotoxic and apoptotic effects [9, 17]. However, in schizophrenia, increased KYNA in the brain may lead to excessive NMDAR blockade, which is a known trigger of psychotic symptoms and cognitive deficits (e.g., with NMDAR antagonists phencyclidine and ketamine) [18].

Increased concentrations of KYN and KYNA have been found in postmortem prefrontal cortex (PFC) brain tissue [19, 20] and cerebrospinal fluid (CSF) [21–23] of people with schizophrenia. Moreover, genetic variants in KP enzymes have been linked to psychotic symptoms, cognitive dysfunction, and abnormal levels of KYNA in schizophrenia [20] and bipolar disorder [24]. This has led to the formulation of the KYNA hypothesis of schizophrenia, centering on the idea that increased KYNA levels cause positive symptoms and cognitive deficits via NMDAR blockade [25]. This is supported by recent studies demonstrating that stress-induced salivary KYNA response in schizophrenia was inversely associated with glutamate levels in schizophrenia [26, 27]. Also, increased intracerebral KYNA in schizophrenia is related to decreased KMO enzyme activity in microglia [20]. However, reduced KMO activity is only indirect evidence of increased KYNA production and KYNA may be expected to be directly related to increased KAT activity since KYNA is produced by KATI/II enzyme activity. Human astrocytes express KATI/II, converting KYN to KYNA, but do not express KMO. Hence, astrocytes are probably the largest producer of KYNA in the brain [28].

Importantly, brain KYNs are linked to and influenced by the peripheral KP [9, 27]. One study indicated that *Toxoplasma gondii* infections enhance production of brain KP metabolites in mice [29], thereby emphasizing the effect of peripheral infections on KYNA levels in the brain. Metabolism in the pathway is driven by blood-derived TRP, KYN, and 3-HK that cross the BBB, whereas KYNA and QUINA cannot cross the BBB due to their polar nature [9]. The breakdown of TRP to KYN reflects the level of activity within the KP; and higher KYN/TRP plasma ratios have been consistently reported in schizophrenia [30–32]. There is a positive association between the proinflammatory cytokine IL-6 and both the KYN/TRP and KYNA/TRP ratios in CSF of patients with schizophrenia [22], and this finding highlights the interaction between activation of inflammation and the KYN/TRP ratio. Furthermore, one recent study showed that increased plasma KYN/TRP levels in schizophrenia were inversely correlated with frontal white matter glutamate levels [30], providing the first evidence that plasma KYN levels can be used as a peripheral indicator of brain changes related to deleterious alterations in cortical neurotransmission. Thus there is evidence supporting the hypothesis that KP metabolites can be used as peripheral markers of brain dysfunction in schizophrenia.

Therefore, we examined postmortem PFC brain tissue from two independent samples of patients and controls and measured brain KP metabolites. We also measured peripheral plasma KP metabolites, cognition, and brain volume in an independent large sample of patients with schizophrenia and healthy controls. Since cytokines can activate the KP, we predicted that increased KP enzyme mRNA should be evident in high cytokine subgroups of schizophrenia. Specifically, we expected to find mRNA levels of TDO and KATI/II mRNAs to be increased in schizophrenia and KMO mRNA to be decreased in schizophrenia, particularly among those patients in the high cytokine subgroup. In addition, we expected to find a higher KYN/TRP ratio, higher KYNA levels, and a higher KYNA/QUINA ratio in postmortem tissue of patients in the high cytokine subgroup. In the cohort of living people, we predicted an increased KYN/TRP ratio in the plasma of patients with schizophrenia compared to controls and that the increased ratio would be more pronounced in the high cytokine subgroup of patients. In addition, we expected that the plasma KYN/TRP ratio would be inversely correlated with executive functioning (working memory and attention) and PFC volume of chronically ill patients with schizophrenia who display increased proinflammatory cytokines.

## Materials and methods

### Postmortem cohorts

#### Tissue Resource Center (TRC) cohort

Postmortem brain tissue samples in this cohort were obtained from the New South Wales TRC. Tissue from the dorsal lateral prefrontal cortex (DLPFC) i.e., middle frontal gyrus, was obtained for 37 individuals with schizophrenia and 37 controls (40 subjects from the right and in 34 from the left hemisphere). Fresh frozen tissue was pulverized and total RNA was extracted using Trizol and complementary DNA (cDNA) was synthesized using Superscript III, as described in Weickert et al. [33]. The present study was performed in accordance with the latest version of the Declaration of Helsinki after review by the Human Research Ethics Committee at the University of New South Wales (UNSW), (HREC #12435).

#### Stanley Medical Research Institute (SMRI) cohort

This cohort includes brain tissue of the middle frontal gyrus from 34 schizophrenia patients and 35 controls received from SMRI. Total RNA from postmortem brain tissue samples were obtained from the SMRI. Tissue characteristics, total RNA extraction, and cDNA synthesis details are provided elsewhere [4]. The present study was performed in accordance with the latest version of the SMRI ethics (http://www.SMRIresearch.org/brain-research/).

#### Quantitative real-time PCR (qPCR)—postmortem cohort

The normalized mRNA expression of KAT I, KAT II, TDO, and KMO, IL-1β, IL-6, IL-8 and Serpin Family A Member 3 (SERPINA3) in the human DLPFC was measured by reverse transcriptase–qPCR (7900HT, Applied Biosystems, Foster City, CA, USA) using pre-designed Taqman Gene Expression Assays in both cohorts. The transcripts for glial fibrillary acidic protein (GFAP) and ionized calcium-binding adapter molecule 1 (IBA1) were also measured in the TRC cohort. For quantitation via qPCR, we used four mRNAs including ACTB (Hs99999903_m1), GAPDH (Hs99999905_m1), TBP (Hs00427621_m1), and UBC (Hs00824723_m1) as normalizing genes. For more details, see Supplemental Methods: Quantitative real-time PCR postmortem cohort.

#### KYN metabolite assays—postmortem cohort

KP pathway metabolites (TRP, KYN, KYNA, QUINA) were assayed from brain tissue, PFC, of the TRC cohort (total *n* = 74) using ultra-high-performance liquid chromatography and gas chromatography–mass spectrometry. Results were normalized to brain tissue mass. For details, see Supplemental Methods: Kynurenine Pathway Metabolite assays – Postmortem cohort.

### Living cohort

#### Participants

Ninety-six chronically ill patients meeting the Diagnostic and Statistical Manual of Mental Disorders, Fourth edition (DSM-IV) criteria for schizophrenia or schizoaffective disorder on the basis of the Structured Clinical Interview for DSM-IV Axis 1 disorders [34] and 81 healthy adults were recruited from two sites (Adelaide and Sydney). For details, see Supplemental Methods: Participants – Living cohort.

All procedures were approved by the UNSW (07–121, 09–187) and South-Eastern Sydney and Illawarra Area Health Service (07–259) Human Research Ethic Committees, Sydney and the Queen Elizabeth Hospital (2010188) Ethics and Human Research Committee, Adelaide. Written informed consent was obtained from each participant before entry into the study.

#### KYN metabolite assays—living cohort

Blood plasma samples were collected from each participant between 8 and 11 a.m. on the day of the magnetic resonance imaging (MRI) scan and cognitive assessments. Assays of TRP, KYN, KYNA, 3-HK, and QUINA were performed as described previously [35]. In addition, C-reactive protein (CRP) was measured in plasma of the living cohort, which is a good indicator for central inflammation [36]. For details, see Supplemental Methods: Kynurenine Pathway Metabolite assays – Living cohort, CRP assays – Living cohort.

#### Cognitive assessments—living cohort

Cognitive domains of verbal memory, language, working memory, processing speed, and perceptual organization were formed as described previously [37]. For details, see Supplemental Methods: Cognitive testing and cognitive domain formation – Living cohort.

#### Gray matter volume data collection and processing—living cohort

MRI was performed on a subset from the whole living sample consisting of 62 patients and 62 controls using a 3-T Phillips Achieva MRI scanner at Neuroscience Research Australia, Randwick, Australia. DLPFC was chosen as the region of interest as prior studies have shown that PFC volume is commonly abnormal in schizophrenia [20, 38–41]. DLPFC was also selected to correspond to regions sampled in the postmortem cohort tissue that included the rostral middle frontal gyrus, bilaterally. For details, see Supplemental Methods: Gray Matter volume data collection and processing—living cohort.

### Cytokine subgrouping

Given that the aim of this work was to determine whether altered KP metabolites existed in subgroups of people with schizophrenia who were classified on the basis of elevated/normal peripheral cytokine levels, our primary analyses were based on our previously established elevated and normal cytokine subgroups [2, 4, 5, 42].

#### Cytokine subgrouping—postmortem cohorts

The low/high cytokine subgroups in the postmortem cohort controls and schizophrenia patients have been described in more detail previously [2, 4]. Preparation of cDNA and qPCR experiments were performed as described previously [2, 4, 42]. From brain tissue cDNA, we assayed for the mRNA levels of: SERPINA3 (Hs003153674_m1), IL-6 (Hs00174131_m1), IL-6ST (Hs01006741_m1), IL-8 (HS00174103_m1), IL-1β (Hs01555410_m1), NFKB1 (Hs00765730_m1), and PTGS2 (Hs00153133_m1) (Applied Biosystems, Foster City, CA, USA)]. These targets were chosen as being most indicative of neuroinflammation in people with schizophrenia from a larger RNA sequencing database. To identify inflammatory subgroups based on cytokine mRNA expression, a recursive two-step cluster analysis was performed on postmortem cohorts initially using all 7 transcripts as input variables as previously described [2, 4]. Four inflammatory markers (SERPINA3, IL-6, IL-1β, IL-8 mRNAs) predicted inflammatory cluster membership with a high degree of confidence, and thus the expression levels of all 4 mRNAs were used to define the high cytokine subgroups as generated by the computer algorithm.

#### Cytokine subgrouping—living cohort

Transcript levels were measured by qPCR using Applied Biosystems’ 7900HT Real-time PCR system (Foster City, CA, USA). Pre-designed Taqman Gene Expression Assays: Interleukin-1β (Hs01555410_m1), Interleukin-6 (Hs00174131_m1), Interleukin-8 (HS00174103_m1), and Interleukin-18 (Hs01038788_m1) (Applied Biosystems, Foster City, CA, USA) were used to quantify the expression of the cytokine genes in the periphery. Three housekeeper genes: Peptidylprolyl isomerase A (Hs99999904_m1), TATA box binding protein (Hs00427620_m1), and ubiquitin C (Hs00824723_m1), were used for normalization of the data. A two-step cluster analysis was performed on all samples in the cohort combined. In the living cohort, four proinflammatory cytokines were measured based on the overlap with the informative cytokines from the brain. We found that four cytokine mRNAs (IL-18, IL-1β, IL-6, IL-8 in order of contribution) significantly contributed to the clustering model (≥0.33 on a scale from 0.1 to 1.0) [42]. Based on these results, patients and controls of both cohorts were separated into high and low cytokine subgroups. For furthers details, see Supplemental Methods: Cytokine subgrouping-living cohort.

### Analyses

#### Statistical analyses: postmortem cohort

All analyses were performed using SPSS (version 25, IBM, Armonk, NY, USA). See Supplemental Table S1 for an overview of the assumptions met in association with the statistical tests related to the groups and metabolites analyzed. Group outliers were identified by Grubb’s test and excluded. Owing to low sample size in the TRC (*n* = 4) and SMRI (*n* = 9) control high cytokine subgroups, these groups were not included in the postmortem cytokine inflammation stratified subgroup analyses. However, the results for these subgroups are included in Supplemental Figure S1 provided for reference purposes. SMRI cohort KP enzyme expression levels were normalized to the TRC cohort healthy control low cytokine subgroup means to combine the two cohorts. Diagnostic differences of KP enzyme mRNA expression between schizophrenia patients and controls in postmortem brain tissue cohorts were analyzed separately and combined. To determine differences among cytokine subgroups, analyses of covariance (ANCOVAs) were performed with KP enzyme mRNA expression and KP metabolite levels and ratios as dependent variables and inflammatory subgroups (those patients with schizophrenia who had high proinflammatory cytokines, normal proinflammatory cytokines and healthy controls with normal proinflammatory cytokine levels) as the grouping variables, with age, RNA integrity number (RIN), or postmortem interval (PMI) as a covariate as appropriate and follow-up post hoc least significant difference (LSD) tests for significant overall ANCOVA results. As an altered inflammation/KP would lead to altered pH values, pH was not used as a covariate. We preformed correlations of KAT I, KAT II, and KMO mRNAs with GFAP and IBA1 mRNAs in the TRC cohort using Spearman correlations.

In the TRC cohort, differences in KP metabolite levels/ratios (KYN/TRP ratio, KYNA, KYNA/QUINA ratio) among cytokine subgroups were analyzed by analyses of variance (ANOVAs)/ANCOVAs with KP metabolites as the dependent variable and inflammatory subgroups as grouping variables with age and PMI as covariates as appropriate and follow-up post hoc LSD tests for significant overall ANOVA/ANCOVA results. All statistical tests were considered significant at *p* < 0.05, 2-tailed, and are reported both as uncorrected and false discovery rate (FDR) corrected. For more details, see Supplemental Methods: Statistical Analyses-postmortem cohort.

#### Statistical analyses: living cohort

Differences in KP metabolites between healthy controls and people with schizophrenia were tested using a series of six univariate ANCOVAs with diagnosis as fixed factor, age as a covariate, and with the five KP metabolites (TRP, KYN, 3-HK, KYNA, QUINA) and the KYN/TRP ratio as dependent variables. To determine the relationship of KP metabolites to cognitive performance and brain volumes, we focused solely on the KYN/TRP ratio given: (a) proinflammatory cytokines directly influence the KYN/TRP ratio by driving the metabolism of TRP to KYN via activation of IDO/TDO [9], (b) previously reported findings of an increased KYN/TRP ratio in schizophrenia [31, 32], and (c) the relationship of the KYN/TRP ratio to brain glutamate levels in schizophrenia [30]. Differences of the KYN/TRP ratio among people with schizophrenia and healthy controls (separated for low and high cytokine subgroups) were tested using an ANCOVA with diagnosis and inflammation status (controls in the low and high cytokine subgroups and people with schizophrenia in the low and high cytokine subgroups) as grouping factors and age as a covariate as appropriate, with follow-up post hoc LSD tests for significant overall ANCOVA results. Pearson correlations of the brain volume region of interest (rostral middle frontal gyrus) and the KYN/TRP ratio were performed in each group (low and high cytokine healthy control subgroups, low and high cytokine schizophrenia subgroups separately). As the DLPFC is involved in attention and working memory, we also performed Pearson correlations between tests of these two cognitive domains and the KYN/TRP ratio in healthy controls and in both the low cytokine and high cytokine schizophrenia subgroups separately. Statistics were performed using SPSS 23.0. All statistical tests were considered significant at *p* < 0.05, 2-tailed. Results are reported as uncorrected and FDR corrected. For more details, see Supplemental Methods: Statistical Analyses – Living cohort.

## Results

### Postmortem cohort

We provide cohort demographics for our postmortem brains on factors including gender, age, tissue pH, RINs, and PMIs (Supplemental Results and Tables S2a and S2b). We detected a few significant correlations of KP metabolites and mRNA expression with demographic variables (Table S3) and with other potentially confounding factors (e.g., medication) (Table S3c). We report group comparisons on the basis of sex (Table S4) and smoking history (Table S5).

### Increased TDO, KAT I, and KAT II mRNA levels in the PFC of a high cytokine schizophrenia subgroup

**Fig. 2:**
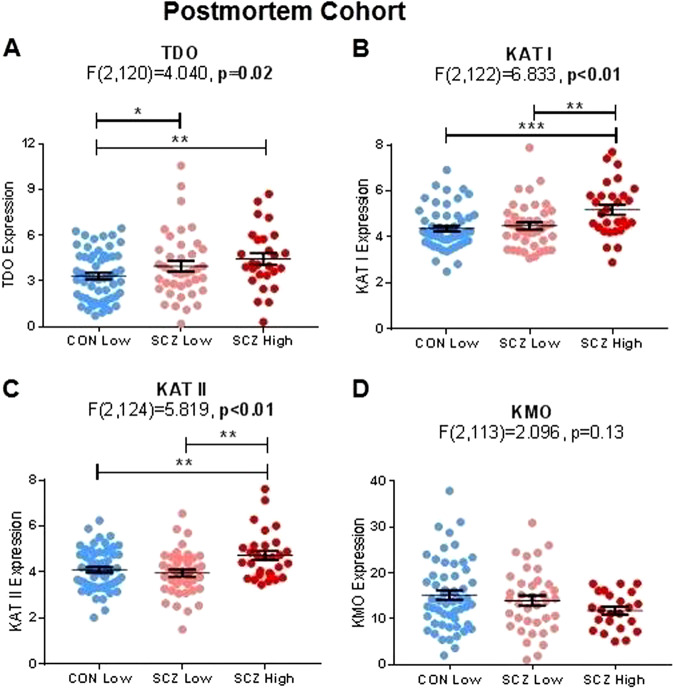
Inflammatory subgroup differences of kynurenine pathway enzyme mRNA expression in New South Wales Tissue Resource Centre (NSW TRC) and Stanley Medical Research Institute (SMRI) combined postmortem brain tissue. **a** Tryptophan-2,3-dioxygenase (TDO) mRNA expression was significantly greater in people with schizophrenia who were in the high cytokine subgroup (*p* = 0.02). **b** Kynurenine aminotransferase (KAT) I mRNA expression was significantly greater in people with schizophrenia who were in the high cytokine subgroup (*p* = 0.01) than other groups. **c** Similarly, KAT II mRNA expression was significantly greater in people with schizophrenia who were in the high cytokine subgroup (*p* = 0.01) than other groups. **d** No statistically significant differences in kynurenine 3 monooxygenase (KMO) mRNA expression were detected among groups. *p < 0.05, **p < 0.01, ***p < 0.001

As both postmortem cohorts showed increased KAT mRNA in high cytokine schizophrenia (Supplemental Figure S1), the cohorts were combined for further analyses to increase power. In the PFC, we found significant differences in brain TDO mRNA among cytokine subgroups (*F*_(2,120)_ = 4.04, *p* = 0.02, FDR *p* = 0.03). Follow-up, post hoc LSD tests showed that TDO expression was significantly increased by 14% (*p* = 0.05) in the low cytokine schizophrenia subgroup relative to normal cytokine controls and increased by 26% (*p* = 0.01) in the high cytokine schizophrenia subgroup relative to normal cytokine controls (see Fig. 2a). Similarly, we found significant differences in KAT I mRNA among cytokine subgroups (*F*_(2,122)_ = 6.83, *p* < 0.01, FDR *p* = 0.02). Follow-up, post hoc LSD tests showed that KAT I mRNA levels were significantly increased by 16% (*p* < 0.01) in the high cytokine schizophrenia subgroup relative to the low cytokine schizophrenia subgroup and increased by 19% (*p* < 0.0001) in the high cytokine schizophrenia subgroup relative to normal cytokine controls (see Fig. 2b). We also found significant differences in KAT II mRNA among cytokine subgroups (*F*_(2,124)_ = 5.82, *p* < 0.01, FDR *p* = 0.02). Follow-up, post hoc LSD tests showed that KAT II mRNA was significantly increased by 19% (*p* < 0.01) in the high cytokine schizophrenia subgroup relative to the low cytokine schizophrenia subgroup and increased by 15% (*p* < 0.01) in the high cytokine schizophrenia subgroup relative to normal cytokine controls (see Fig. 2c). There were no significant differences among cytokine subgroups in relation to KMO mRNAs (see Fig. 2d). Additional information on enzyme mRNA levels on the basis of diagnostic groups (Supplemental Figure S2) or diagnostic inflammation subgroup in NSW TRC and SMRI cohorts separately can be found in Supplemental Results (Supplemental Figure S1).

### GFAP and IL-6 mRNAs correlate with mRNAs encoding KAT I and II enzymes in the PFC of schizophrenia patients

**Fig. 3:**
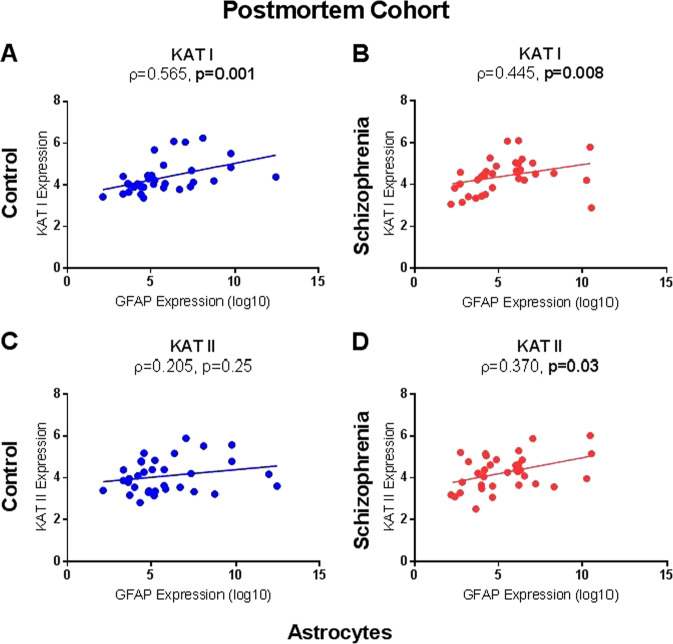
Correlations of kynurenine aminotransferases I (**a**, **b**) and II (**c**, **d**) (KATI/II) mRNA expression with glial fibrillary acidic protein (GFAP) in postmortem brain tissue. KATI mRNA expression significantly correlates with GFAP mRNA in **a** controls (*p* = 0.001) and **b** schizophrenia (*p* = 0.008), whereas a significant correlation for KATII was only found in **d** schizophrenia patients (*p* = 0.03)

A moderately strong, significant, positive correlation was found between an astrocyte maker (GFAP) and KAT I mRNAs in both healthy controls (*ρ* = 0.57, *n* = 33, *p* = 0.001, FDR *p* = 0.004, see Fig. 3a) and in patients with schizophrenia (*ρ* = 0.45, *n* = 36, *p* < 0.01, FDR *p* = 0.02, see Fig. 3b). A similar but slightly weaker correlation was found between GFAP and KAT II mRNAs in patients with schizophrenia (*ρ* = 0.37, *n* = 36, *p* = 0.03, FDR *p* = 0.04, see Fig. 3d); however, there was no significant correlation between GFAP and KATII mRNAs in controls (*ρ* = 0.21, *n* = 34, *p* = 0.25, FDR *p* = 0.25, see Fig. 3c). No significant correlations were found between a microglial marker (IBA1) and KMO mRNAs in either patients with schizophrenia or healthy controls.

In the TRC cohort, there were moderately strong, positive, significant correlations between KAT I/II mRNAs and many cytokines assessed (IL-1β, IL-6, and IL-8) and SERPINA3 in schizophrenia and control groups (Supplemental Table S6). In contrast, we detected moderately strong, significant inverse correlations of KMO mRNA levels to IL-1β and SERPINA3 mRNA levels (see Supplemental Table S6) within the PFC of people with schizophrenia. TDO mRNA did not show any significant correlations with any of the cytokine mRNAs assayed. There were also moderately strong, significant correlations of KATI mRNA with IL-6 and SERPINA3 mRNAs in people with schizophrenia in the SMRI cohort (Supplemental Table S6b).

### Increased KYN/TRP ratio and increased KYNA in the PFC of a high cytokine schizophrenia subgroup

**Fig. 4:**
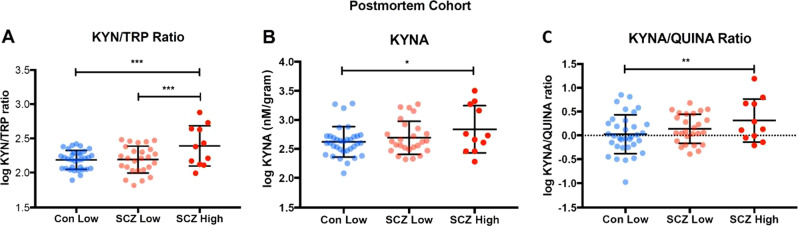
Inflammatory subgroup differences of kynurenine (KYN) pathway metabolites in postmortem brain tissue. **a** KYN/tryptophan ratio was significantly greater in people with schizophrenia who were in the high cytokine subgroup (red, *p* < 0.001) compared to both low cytokine controls (light blue) and low cytokine schizophrenia (pink) subgroups. **b** Kynurenic acid (KYNA) was significantly greater in people with schizophrenia who were in the high cytokine subgroup (red) compared to the low cytokine controls (light blue) (*p* = 0.013). **c** KYNA/quinolinic acid ratio was significantly greater in people with schizophrenia who were in the high cytokine subgroup (red) as compared to the normal cytokine control group (light blue, *p* = 0.01). *p < 0.05, **p < 0.01, ***p < 0.001

In the PFC, we found significant differences in the KYN/TRP ratio among cytokine subgroups (*F*_(2,66)_ = 8.25, *p* = 0.001, FDR *p* = 0.003). Follow-up, post hoc LSD tests showed that the KYN/TRP ratio was significantly higher in the high cytokine schizophrenia subgroup relative to normal cytokine schizophrenia subgroup of patients (*p* < 0.001) and relative to normal controls (*p* < 0.001, Fig. 4a).

Further, we found significant differences in KYNA among cytokine subgroups (*F*_(2,66)_ = 3.37, *p* = 0.04, FDR *p* = 0.04). Follow-up, post hoc LSD tests showed that higher KYNA was found in the high cytokine schizophrenia subgroup relative to normal cytokine controls (*p* = 0.013), whereas no other significant subgroup differences were detected (Fig. 4b).

Finally, a significant difference was also found in KYNA/QUINA ratio among cytokine subgroups (*F*_(2,66)_ = 3.53, *p* = 0.04, FDR *p* = 0.04). Follow-up, post hoc LSD tests showed that higher KYNA/QUINA ratio (Fig. 4c) was found in the high cytokine schizophrenia subgroup relative to normal cytokine controls (*p* = 0.01), whereas no other significant differences were detected.

### Living cohort

Patient and control groups were not significantly different on the basis of sex ratios and both groups were in their early to mid-30s on average in relation to age (Supplemental Table S2c). As expected, the patients differed significantly from healthy controls on the basis of demographic, cognitive, and brain volume variables (Supplemental Table S7), but there were few significant correlations between KYN/TRP ratio and demographic variables in patients and controls or with clinical variables in patients (Supplemental Table S8), and no significant differences in KP metabolites on the basis of serotonin-altering medication (Supplemental Table S9) or smoking status (Supplemental Table S10) in patients. Attention was related to KYN/TRP ratio and PANSS negative symptom severity in all patients (Supplemental Table S11), and there was a significant difference between high and normal cytokine patient groups on the basis of reading and attention (Supplemental Table S12). There were some significant, positive correlations between cytokines and KYN/TRP ratio (Supplemental Table S13).

### Decreased plasma TRP and KYNA but increased KYN/TRP ratio in schizophrenia

People with schizophrenia had significantly lower levels of plasma TRP (*F*_(1,174)_ = 17.9, *p* < 0.001, FDR *p* = 0.003) and plasma KYNA (*F*_(1,175)_ = 20.1, *p* < 0.001, FDR *p* = 0.003) but a significantly higher KYN/TRP ratio (*F*_(1,175)_ = 5.1, *p* = 0.025, FDR *p* ≤ 0.05), relative to healthy controls (Supplemental Figure S3). KYN/TRP ratio correlated significantly with CRP (r = 0.28, *p* = 0.01) and IL-1β mRNA (r = 0.23, *p* = 0.03) in schizophrenia patients but not in controls (CRP r = 0.13, *p* = 0.28; IL-1β mRNA, r = −0.14, *p* = 0.26, Supplemental Table S13). There were no other significant correlations of the KYN/TRP ratio to other cytokine mRNAs (IL-6, IL-8, IL-18, all *p*s > 0.05, Supplemental Table S13). There were no other significant differences in plasma KP metabolite levels (KYN, *F*_(1,174)_ = 2.7, *p* = 0.07, FDR *p* = 0.1; 3-HK, *F*_(1,175)_ = 1.4, *p* = 0.25, FDR *p* = 0.28; QUINA, *F*_(1,172)_ = 1.2, *p* = 0.28, FDR *p* = 0.28) between patients and controls (see Supplemental Figure S3).

### Plasma KYN/TRP ratio is highest in a high cytokine schizophrenia subgroup

**Fig. 5:**
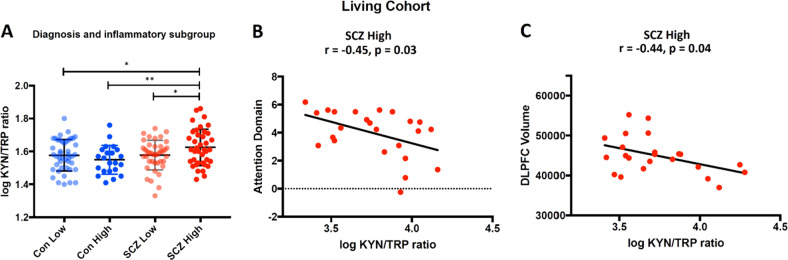
Elevated kynurenine (KYN)/tryptophan (TRP) ratio is related to dorsolateral prefrontal cortex (DLPFC) volume and attention in patients with schizophrenia (SCZ) who display elevated peripheral cytokine levels. **a** Comparison of plasma KYN/TRP ratio in patients with schizophrenia who display high cytokines levels to patients with SCZ who display low cytokine levels and healthy controls with high and low cytokine levels. Patients with SCZ who display elevated proinflammatory cytokines (high) displayed significantly elevated KYN/TRP ratio compared to patients with schizophrenia who displayed low cytokine levels (SCZ low) and healthy controls who displayed low (Con low) and high (Con high) cytokine levels. *p < 0.05, **p < 0.01. **b** In SCZ patients with high proinflammatory cytokines, a significant negative correlation was detected between KYN/TRP ratio and the attention domain, indicating worse attention performance with elevated KYN/TRP ratio. **c** In SCZ patients with high proinflammatory cytokines, a significant negative correlation was detected between KYN/TRP ratio and dorsolateral prefrontal cortex (DLPFC) volume

We found significant differences in KYN/TRP ratio among diagnostic groups categorized on the basis of peripheral cytokine levels (*F*_3,143_ = 3.3, *p* = 0.02, FDR *p* = 0.02). Follow-up LSD pairwise comparisons demonstrated significantly increased KYN/TRP ratio in the high cytokine schizophrenia subgroup as compared to low cytokine controls (*p* = 0.03), high cytokines controls (*p* = 0.004), and the low cytokine schizophrenia subgroup (*p* = 0.03, see Fig. 5a). No other KP metabolites showed significant differences based on cytokine subgroups (all *t*s < 1.0, all *p*s > 0.05).

### Attention performance and DLPFC volume correlated inversely with plasma KYN/TRP ratio in patients with schizophrenia who had high cytokine levels

There were moderately strong, inverse, significant correlations of KYN/TRP ratio to attention (*r* = −0.45, *p* = 0.03, *n* = 24) and rostral middle frontal gyrus volume (*r* = −0.44, *p* = 0.04, *n* = 23) in the high cytokine subgroup of patients with schizophrenia (see Fig. 5b, c). Thus, as KYN/TRP ratio increased, attention and PFC volume decreased. No significant correlation was found between KYN/TRP ratio and working memory (*r* = 0.12, *p* = 0.47). There were no significant correlations of brain (DLPFC) volumes or cognition (working memory, attention) with the KYN/TRP ratio in the low cytokine schizophrenia subgroup or low and high cytokine healthy controls (all *r*s ≤ 0.30, all *p*s > 0.05).

## Discussion

Our results support the involvement of the immune-activated KP of TRP metabolism in the pathophysiology of a subset of people with schizophrenia having elevated cytokines from five distinct aspects: (1) elevation of KP enzyme mRNAs in human brain, (2) alterations of KP metabolites in human brain, (3) peripheral changes in KP metabolites, (4) peripheral KP measures that link with cognitive deficits, and (5) peripheral KP measures that link with structural human brain volumetric abnormalities. In postmortem tissue, we showed that the mRNA for the KYN-producing enzyme, TDO, and the KYNA-producing enzymes, KATI/II, are increased in patients with schizophrenia who also display elevated cytokine levels. Activation of the KAT arm of KP metabolism in the brain is likely through astrocytes, since KAT is localized to astroglia and KAT mRNAs positively correlated with the astroglial marker, GFAP mRNA, in our sample, suggesting co-regulation. Our observations are consistent with a model of pathophysiology whereby a cytokine-induced intracerebral overproduction of KYNA via KAT in astrocytes occurs in at least some people with schizophrenia. Since KYNA functions as an NMDA and alpha 7 receptor antagonist [9], higher KYNA may provide a direct link to cortical changes capable of yielding cognitive disruptions.

In accordance with our results on KP enzyme mRNA showing increased KAT I and KAT II mRNA, we find significant elevations of the KYN/TRP ratio and KYNA levels in postmortem brain tissue of schizophrenia patients with high proinflammatory cytokines. Thus the present study confirms a link between inflammation markers and KP activation—to our knowledge for the first time—within the brains of patients with schizophrenia.

Consistent with the findings in postmortem brain tissue, the KYN/TRP ratio was also increased in the plasma of people with schizophrenia who had elevated cytokine levels from the living cohort. The increased breakdown of TRP to KYN points toward a cytokine-driven upregulation in the KP in schizophrenia periphery and brain and is in line with several previous studies [22, 30–32]. In support of the hypothesis regarding inflammation in the brains of patients with schizophrenia, we have previously demonstrated that significantly more patients with schizophrenia as compared to controls have a high inflammatory biotype in independent postmortem cohorts [2, 4] that has also been independently replicated in peripheral blood from a living cohort [5, 42]. However, increases in circulating ratios of KYN/TRP may or may not have a direct impact on the biochemistry and/or function of the human brain. In support of an effect, we report the first evidence to link increased plasma KYN/TRP ratio with reduced DLPFC volume and attention in people with schizophrenia displaying increased proinflammatory cytokines. Also, associations between a higher KYN/TRP ratio and cognitive and structural abnormalities mimicking NMDA-related deficits would be consistent with increased brain KYNA [30, 43]. Interestingly, reductions of brain KYNA via a KATII inhibitor have produced improvements of attention in rodents [44]. KYN and TRP both cross the BBB and can therefore influence brain levels [35]. Especially in conditions of systemic inflammation, the majority of brain KYN is derived from blood [9]. Moreover, our data suggest that plasma levels of KYN/TRP can provide a readily measureable peripheral indicator of the degree of attention impairment and DLPFC brain volume loss in people with schizophrenia who also display increased peripheral cytokine levels. However, studies are needed measuring KP enzyme activity and metabolites in plasma and CSF of the same patients to further support these findings.

In contrast to the putative overproduction of KYNA in the brain, our current finding supports other work [45–48] showing reduced TRP and KYNA in circulation of people with schizophrenia relative to controls. However, plasma KYNA cannot cross the BBB and might therefore be decoupled from brain KYNA in schizophrenia. While previous studies [45, 46, 49] have interpreted that the lower blood levels of the proposed “neuroprotective” KYNA may lead to schizophrenia-like changes due to putatively increased “neurotoxic” QUINA, there is another possible interpretation. We suggest that the lower KYNA levels in circulation may reflect increased exit of TRP and KYN from the blood and more entry into the brain, which could lead to more substrate for brain KP activity and increased KYNA production in the brains of people with schizophrenia. This is consistent with a previous study showing that therapy with the proinflammatory cytokine interferon-alpha in patients with hepatitis C led to increases in KYN and KYNA in CSF, simultaneous with the emergence of psychiatric symptoms and also with concomitant decreases in plasma TRP [50]. However, given that our brain and blood measurements were not obtained from the same individuals, our hypothesis pertaining to differences in the blood and brain levels of TRP and KYNA within the same individuals remains speculative.

Cytokines can activate microglia and astrocytes, which are key players in KP metabolism. KAT I/II mRNA is mainly produced in astrocytes [51] and we find that KATI/II mRNA was increased in the brains of people with schizophrenia who were in the high cytokine subgroup, whereas KMO mRNA was not statistically different according to diagnosis or cytokine status. If the mRNA differences we detected translate to changes in protein levels and enzyme activity in the brain, then increased brain KYN may be biased toward more production of KYNA and not necessarily to more 3-HK or QUINA. Since astrocytes are considered a most important source of KYNA [28], we would expect a relationship between the levels of KATI/II and the astrocyte marker GFAP. Indeed, we previously found increased GFAP mRNA and protein in those patients with schizophrenia who also had elevated cytokines [52] and a positive correlation between KATI/II mRNAs and both GFAP and cytokine mRNAs in the present study. Further, we found that there was a greater likelihood of more reactive astrocyte morphology in the brains of patients with schizophrenia who also had elevated cytokines compared to controls, suggesting that astrocytes may contribute to the elevations in KYNA we detect. However, anatomical mapping the KATI/II mRNA and protein in the brains of people with schizophrenia is necessary to support the cellular source of increased KATI/II.

There are several limitations to this study. Our result of increased KYN in the blood of people with schizophrenia indicates that there may be a possible upregulation of the first step in the peripheral KP via conversion of TRP to KYN. However, we have not directly measured peripheral TDO protein levels or enzyme activity in our study. In fact, the lack of direct activity-level measurements for any of the KP enzymes within the blood and brain is a limitation of our study, and further work is necessary to understand in more detail which aspects of KP regulation are altered. Another limitation is that we sampled from one point in time. Future studies of living patients should address whether the dysregulation of KP metabolites remains stable over time or if the metabolites vary throughout the course of the disease or with acute exacerbations of symptoms. Longitudinal studies will be important if we are to use KP metabolites and immunological factors as clinical biomarkers [53], especially when cytokines are being considered as potential biomarkers aimed at predicting conversion to illness in high-risk samples [54]. A final limitation for our study is that since TRP levels can be influenced by multiple factors (e.g., dietary and lifestyle) we can not rule out that these between groups differences could be derived from potential confounds that were not assessed in our study.

While the high inflammation biotype of schizophrenia is a novel stratification approach based on recent findings, as described above, we have previously reported that a significantly higher percentage of patients with schizophrenia had a high inflammatory biotype in three independent cohorts [2, 4, 5, 42] and others have reported similar findings [55]. We did not explore differences in the high cytokine control group in our postmortem sample due to the small sample size in that subset; however, our living cohort showed significant differences on the KYN/TRP ratio in high inflammation patients vs. each of the other inflammation categories of patients and controls, including high inflammation controls. Future studies should also investigate high cytokine controls as assayed from brain tissue to confirm whether these changes are only found in schizophrenia or are part of a more generalizable inflammatory response. Further, in disease states there may be changes in cell-subtype expression of mRNAs, particularly in schizophrenia. The lack of cellular specificity for the postmortem study is a limitation of the present study.

Also, although we did not show relationships between antipsychotics and KP metabolites in the postmortem or living samples, antipsychotics may be a limiting factor in our study. In addition, although the two postmortem and one living cohorts are all independent samples, we have reported on each of these cohorts to some extent previously in relation to other variables and hypotheses [2, 4, 5, 42]. There are also studies that did not confirm an activation of the brain immune system in schizophrenia [56]. Nevertheless, this report did initially find an increase of IL-6 mRNA in schizophrenia compared to controls (*p* = 0.04, FDR corrected), which was dismissed on the basis of further post hoc statistical procedures (principal component-based correction). We suggest caution should be applied to avoid overcorrection of highly correlated tests in statistical adjustment for cytokine differences between multiple groups in future studies [57] and that a stratification approach may be more powerful in terms of defining neuroimmune-related pathophysiology in the brains of people suffering from schizophrenia. One strength of our present study is that we find KP activation in high cytokine schizophrenia patients in three independent cohorts (TRC, SMRI, Living cohort).

In summary, this is the first study showing alterations in and associations among KP enzyme mRNAs, KP metabolites in the brain and plasma, brain volume, and cognition in subgroups of people with schizophrenia who have elevated inflammation markers. Based on the results of the present study and previous literature, we propose the following model for KP dysregulation in schizophrenia: inflammatory mechanisms induce an increased conversion of TRP to KYN in the periphery (which can cross the BBB) leading to elevated brain KYN that is converted to KYNA by the proinflammatory-driven increase in KAT enzyme activity via increased mRNA in astroglia. This increased KYNA eventually causes brain volume loss and attention impairment via extensive NMDAR blockade. Future studies are needed to confirm this model and to test whether KP metabolites and proinflammatory cytokines in either plasma or CSF in psychosis risk and first-episode psychosis are useful clinical biomarkers for schizophrenia.

## Supplementary information

Supplemental Material
