# Supplementary material for: Dysregulation of kynurenine metabolism is related to proinflammatory cytokines, attention, and prefrontal cortex volume in schizophrenia
Source: Mol Psychiatry. 2019 Apr 3;25(11):2860–72. doi: 10.1038/s41380-019-0401-9 (PMC7577855; doi:10.1038/s41380-019-0401-9)
Supplement: Supplementary file 1 — Supplemental Material [file 41380_2019_401_MOESM1_ESM.docx]

**Supplemental Material:**

**Dysregulation of kynurenine metabolism is related to proinflammatory cytokines, attention and prefrontal cortex volume in schizophrenia.**

Jochen Kindler*^1, 2, 3^, Chai K. Lim*^4^, Cynthia Shannon Weickert^1,2,5^, Danny Boerrigter^2,5^, Cherrie Galletly^6, 7,8^, Dennis Liu^6, 8^, Kelly R Jacobs^4^, Ryan Balzan^9^, Jason Bruggemann^1, 2^, Maryanne O’Donnell^1,10^, Rhoshel Lenroot^1,2,5^, Gilles J. Guilleminꝉ^4^, Thomas W. Weickertꝉ^1,2,5^

^1^ School of Psychiatry, University of New South Wales, Randwick, NSW, 2031 Australia

^2^ Neuroscience Research Australia, Randwick, NSW, 2031 Australia

^3^ University Hospital of Child and Adolescent Psychiatry and Psychotherapy, University of Bern, 3000 Bern, Switzerland

^4^ Department of Biomedical Sciences, Faculty of Medicine and Health Sciences, Macquarie University, NSW, 2109 Australia

^5^ Schizophrenia Research Institute, Randwick, NSW, 2010 Australia

^6^ Discipline of Psychiatry, School of Medicine, University of Adelaide, Adelaide, SA, Australia

^7^ Ramsay Health Care (SA) Mental Health, Australia

^8^ Northern Adelaide Local Health Network, Adelaide, SA, Australia

^9^ School of Psychology, Flinders University, Adelaide, SA, Australia

^10^ Kiloh Centre, Prince of Wales Hospital, Randwick, New South Wales, Australia

*/ꝉ these authors contributed equally to this work

**Supplemental Methods**

**Quantitative real-time PCR - Postmortem cohort**

The mRNA expression of KATI, KAT II, TDO, KMO, IL6 and glial fibrillary acidic protein (GFAP) in the human DLPFC was measured by RT-qPCR (7900HT, Applied Biosystems, Foster City, CA, USA) using pre-designed Taqman Gene Expression Assays, KAT I (Hs00187858_m1), KAT II (Hs00212039_m1), TDO (Hs00194611_m1), KMO (Hs00175738_m1), IL6 (Hs00174131_m1) and GFAP (Hs00909236_m1), (Applied Biosystems, Foster City, CA, USA). Expression levels were normalised to the geometric mean of four housekeeper genes; ACTB (Hs99999903_m1), GAPDH (Hs99999905_m1), TBP (Hs00427620_m1) and UBC (Hs00824723_m1). Housekeeper geomeans did not differ among diagnostic (all p’s > 0.6) or cytokine (all p’s > 0.08) groups.

**Kynurenine Pathway Metabolite assays – Postmortem cohort**

Fresh frozen brain tissues (~100mg) were mechanically homogenised in 500 µL ice-cold 50% v/v acetonitrile using Precellys 24 tissue homogenizer (Bertin Instruments, France) and 2 mL Precellys Lysing kit tubes containing 1.4 mm ceramic beads (Bertin Instruments, France; 6000 RPM for 0.5 min, repeated twice). Brain homogenates were kept on ice and cells were lysed using microprobe sonicator for 0.25 min, (repeated twice) and cellular debris removed by centrifugation at 12 000 g at 4˚C for 15 min. Cell lysates (300 µL) were then dried under vacuum and resuspended in 75 μL distilled water. Remaining soluble proteins were precipitated with the addition of 75 μL 10 % w/v trichloroacetic acid. The resulting supernatants were filtered through 0.22 μm Ultrafree-MC GV centrifugal filters (Merck Millipore, Germany) at 12 000 g at 4˚C for 15 min and analysed for KP pathway metabolites by ultra high performance liquid chromatography (uHPLC) and Agilent 7890A gas chromatography–mass spectrometry (GCMS) as outlined below. Briefly, 200 μL of samples were treated with equal volume of 10% trichloroacetic acid and filtered through a 0.45 μm PTFE syringe filter to isolate the KP metabolites. Treated samples were then injected into a Poroshell RRHT C18 column (Agilent Technologies; 1.8 μm, 100 x 2.1mm i.d.) maintained at 38 °C and an injection volume of 20 μL via an automatic liquid sampler. Results were normalised to brain tissue mass (frozen weight) and expressed as nM/g tissue weight. Experimenters were blind to diagnostic group and inflammatory status throughout these procedures.

**Cytokine subgrouping - Postmortem cohort**

The low/high cytokine sub-groups in the postmortem cohorts controls and schizophrenia patients have been described in more detail previously.^1-3^

**Participants - Living cohort**

All participants were screened for exclusion criteria, which included a concurrent DSM-IV Axis I diagnosis other than schizophrenia or schizoaffective disorder for patients or any personal history or first-degree relative with a DSM-IV Axis I disorder for controls; for all participants: a history of uncontrolled diabetes or cardiovascular disease including hypertension, central nervous system infection, recent alcohol/substance abuse (within the past 5 years), head injury with loss of consciousness, epileptic seizures, structural brain abnormalities, developmental disorders, and/or mental retardation. All people with schizophrenia were receiving antipsychotic medication (95% receiving second-generation antipsychotics) for at least 1 year prior to participation. Mean daily dose of antipsychotic medication for each patient was converted to approximate daily mean chlorpromazine milligram equivalents (CPZ) dose using standard guidelines.^4^ Symptom severity in patients was assessed using the Positive and Negative Syndrome Scale (PANSS)^5^ by a psychologist or psychometrician trained in administration and scoring.

**Kynurenine Pathway Metabolite assays - Living cohort**

Plasma samples were treated with trichloroacetic acid at a final concentration of 5%, centrifuged (4 °C) for 10 min at 12 000 r.p.m and stored at -80 °C. KP metabolites were assayed from plasma using an Agilent 1290 ultra high performance liquid chromatography (uHPLC) system. Assays of TRP, KYN, 3-HK and KYNA were performed as previously described.^6^ QUINA was concurrently measured using an Agilent 7890A gas chromatography–mass spectrometry (GCMS) system. Briefly, 200 μL of samples were treated with equal volume of 10% trichloroacetic acid and filtered through a 0.45 μm PTFE syringe filter to isolate the KP metabolites. Treated samples were then injected into a Poroshell RRHT C18 column (Agilent Technologies; 1.8 μm, 100 x 2.1mm i.d.) maintained at 38 °C and an injection volume of 20 μL via an automatic liquid sampler. The assay was performed using 0.2 mM sodium acetate at pH 4.65 as mobile phase at an isocratic flow of 0.75 mL/min. TRP was quantified by fluorescence while KYN and 3HK were quantified by UV detection (Ex 254/Em 404 and UV/Vis detection at 365 nm, respectively).

Measurement of KYNA was performed as previously described.^6^ Treated samples were injected into a C18 column and KYNA was eluted using a mobile phase consisting of 50mM sodium acetate supplement with 25 mM zinc acetate and 2.25% (v/v) acetonitrile as organic modifier (solvent A) and 10% acetonitrile (solvent B). A 10μL sample volume was used to inject into a Poroshell RRHT-C18 column at an isocratic flow rate of 0.75 mL/min with a gradient elution profile of 100% solvent A for 3 min and then 50% solvent A and 50% solvent B for 2 min, followed by 100% solvent B for 2 min and 100% solvent A (run time 10 min). Quantification of KYNA was carried out by fluorescence (Ex 344/Em 388) detection and expressed in nM. Intra- and inter-assay coefficient of variation (CV) was determined by standards incorporated in the sample run set to be in an acceptable range of 5–8%.

QUINA was measured using an Agilent 7890A gas chromatography coupled with a Agilent 5975C mass spectrometry detector as previously described.^6^ Briefly, 50 μL of treated sample was added with deuterated internal standards and dried completely in a vacuum concentrator (Thermo SpeedVac). The solutes were derivatised with trifluoroacetic anhydride and hexafluoroisopropanol before dissolving in toluene. The final product was filtered with silane treated glass wool and anhydrous sodium sulphate before injecting (1 μL) into a gas chromatography column (Agilent J&W, DB-5MS) with assay conditions in accordance to the method described previously.^7^ Results were recorded in nM. The intra- and inter-assay CV was determined by standards incorporated in the sample run set to be in the acceptable range of 7–10%.

**C-Reactive Protein assays – Living cohort**

Peripheral venous blood was collected from all participants in 9ml ethylenediaminetetraacetic acid (EDTA) tubes (Vacuette Vacutainer, Greiner Bio-One, Kremsmünster, Austria). Plasma was collected from EDTA tubes via centrifugation for 15 minutes at 2000 x g. Plasma was then aliquoted into protein low-binding tubes (Eppendorf, Hamburg, Germany) and stored at -80 degrees celsius until the day of the assay.

CRP was measured in plasma using a high-sensitivity Enzyme-linked immunosorbent assay (hsCRP, ELISA) according to the manufacturer’s instructions (IBL-international, Hamburg, Germany). Ten microliters of plasma was diluted serially to a final dilution of 1:1000 and each assay was run back to back with samples in duplicate by the same investigator, who was blind to the diagnosis. A five-point standard curve was generated using 0 µg/ml, 0.4 µg/ml, 1 µg/ml, 5 µg/ml and 10 µg/ml calibrators that were prepared by the manufacturer. The average coefficient variance across all plates was 3.23%. The sample reads ranged from 0.06 mg/L to 23.92 mg/L. A minimum detectable value replacement of 0.003 was used for 13 data points that were below the respective minimal detectable value on all protein analytes.^8^

**Cognitive testing and cognitive domain formation – Living cohort**

All participants were administered a four subtest version of the Wechsler Adult Intelligence Scale-Third Edition (WAIS-III)^9^ (consisting of Arithmetic, Similarities, Picture Completion and Digit Symbol Substitution subtests) as an estimate of current IQ and the Wechsler Test of Adult Reading (WTAR)^10^ as an estimate of premorbid intelligence in patients. The WAIS-III Letter-Number Sequencing (LNS) test was included to assess working memory^9^. Both the immediate Logical Memory I (LM I) and delayed Logical Memory II (LM II) verbal memory tests of the Wechsler Memory Scale-Revised (WMS-R)^11^ were administered to assess immediate and delayed verbal memory and the Controlled Oral Word Association Test (COWAT) F-A-S verbal fluency test was used to assess language ability.^12^ Form A of the Trail Making Test (TMT-A)^13^ was used to assess attention. All tests were administered by a trained research assistant or psychologist.

Where possible age scaled scores (SS) for cognitive variables were used for analyses. Age SS were calculated for all WAIS-III subtests and COWAT scores were adjusted for sex and years of education. Raw scores were used for LMI and LMII. The scores were next converted to Z-scores which were then grouped into the following five cognitive domain scores: verbal memory (WMS-R LMI and LMII), language (COWAT FAS and WAIS-III similarities), working memory (WAIS-III arithmetic and LNS), processing speed (WAIS-III DSST), and perceptual organization (WAIS-III Picture Completion) to reduce the number of variables for statistical analyses.^14^ When more than one test was used for a given cognitive domain score the Z-scores for each variable were summed to provide a composite score. An additional domain of attention, Form A of the Trail Making Test (TMT-A duration), was added in a subset of the sample. TMT-A z-scores were inverted (Table S7) and a constant (c = 6) was added for visualization of the correlation results (Figure 5B). Thus, higher TMT-A values in figure 5 B indicate better performance on the attention task.

**Gray Matter volume data collection and processing – Living cohort**

We obtained a T1-weighted, high- resolution anatomical scan with an 8 channel bird cage head coil for each participant, repetition time (TR) 5.4 ms, echo time (TE) 2.4 ms, field of view (FOV) 256 mm, matrix 256 × 256, sagittal plane, slice thickness 1 mm, no gap, 180 slices. All data sets were screened for anatomical abnormalities, excessive motion, successful normalization and artifacts. MRI image analysis was performed on a Mac OSX 10.8 workstation using FreeSurfer version 5.1. software. Images were next run through a first-level autoreconstruction in FreeSurfer. The skull-stripped brains were checked for remaining dura, sinuses or other artifacts that could interfere with successful segmentation. When artifacts were found, images were edited manually. When deemed sufficiently clean for segmentation, images were run through second- and third-level autoreconstruction, in which gray matter surface area, thickness, and volume measures were extracted. Finally, automated cortical parcellation was performed using a separate processing pipeline included in the FreeSurfer software package. All individuals with MRIs were available for our analysis, except three individuals (1 control, 2 patients) that were excluded due to structural MRI abnormalities, leaving 60 patients and 61 controls for the MRI analysis. However, all three measures (MRI, KYN/TRP ratio, and proinflammatory cytokines) were available for only 50 patients and 51 controls for the final MRI analysis. No further subjects were excluded.

**Cytokine subgrouping - Living cohort**

Missing values were replaced by an expectation maximization algorithm for the four pro-inflammatory cytokines (IL-1β, IL-6, IL-8, IL-18).^3^ Only individuals with gene expression data on at least three out of four inflammatory markers were included in the expectation maximization algorithm, others were excluded (n=4).^3^ No RNA/cDNA was available for 15 participants; therefore, no cytokine gene expression could be measured in those individuals. Cytokine values > 2 standard deviations from the group mean were considered outliers. Individuals were also excluded from clustering if two or more cytokines were outliers (7 patients, 4 controls). The final sample (n=147) consisted of 45 healthy controls with low inflammation, 22 healthy controls with high inflammation, 41 schizophrenia patients with low inflammation and 39 schizophrenia patients with high inflammation.

**Analyses**

**Statistical Analyses: Postmortem cohort**

Group outliers were identified by Grubb’s test and excluded. The mRNA expression levels were normally distributed for KAT I and KMO, whereas normal distribution was achieved after log transformation for GFAP and IL6. KYN/TRP ratio, KYNA and KYNA/QUINA ratio levels where normally distributed after log transformation. Correlations of KYN pathway enzyme mRNA expression levels with postmortem interval (PMI), RNA integrity number (RIN), pH and age were performed in controls and schizophrenia patients separately. Correlations of KYN pathway enzyme expression levels with duration of illness and daily and lifetime chlorpromazine equivalents dose were performed in schizophrenia patients only. To determine sex differences in these metabolites/mRNA expression, t-tests were performed between male and female participants in patient and control groups combined.

As an altered inflammation/KP could lead to lower brain tissue pH values, using pH as a covariate may inappropriately remove too much disease relevant variance and impair our ability to detect diagnostic and inflammation-related brain change, thus pH was not used as a covariate in our analyses.

**Statistical Analyses: Living cohort**

KYNA, QUINA, 3-HK and KYN/TRP ratio data were log transformed. After log transformation, parametric distributions were achieved for all KP metabolites with the exception of TRP in controls. A total of 11 participants (7 patients and 4 controls) were excluded (due to cytokine values > 2 SD from the mean) prior to the cluster analysis and only one patient was excluded from our analyses of interest due to an elevated CRP level, suggesting acute bacterial infection.

Since serotonin-altering medications (SAMs) might influence KP plasma metabolite levels, t-tests were performed in the patient group between those patients receiving SAMs (i.e., Selective Serotonin Reuptake Inhibitors, Serotonin-Norepinephrine Reuptake Inhibitors, Noradrenergic and Specific Serotonergic Antidepressants) and those patients who were not receiving SAMs on all KP plasma metabolites.

Correlations were performed between the KYN/TRP ratio variable and all demographic variables in patient and control groups separately. Correlations were also performed between KYN/TRP and illness duration, age of illness onset, mean daily CPZ (mg) and imipramine equivalent dose in the patient group only.^4, 15, 16^ To determine potential sex and smoking differences in KYN/TRP ratio, t-tests were performed between sexes in patient and control groups separately and on the basis of smoking/non-smoking in patients only.

**Supplemental Results**

**Demographics and clinical characteristics - Postmortem cohorts**

There were no significant differences between patients and controls in relation to the demographic variables of age, sex, PMI or RIN (Table S2a, S2b). There was a significant difference in pH levels between patients and controls only in the SMRI cohort. In general, the patients were chronically ill and had been receiving antipsychotics (Table S2a, S2b).

**KP enzymes and their associations with potentially confounding factors – Postmortem cohort**

In the New South Wales Tissue Resource Centre (TRC) cohort, there were moderately strong, significant correlations between KATI mRNA expression and age and KATII mRNA expression and pH in patients (see Table S3A). KATI mRNA expression correlated with duration of illness, lifetime and daily chlorpromazine^4^ (CPZ) equivalent dose, and KMO mRNA expression correlated with mean daily CPZ equivalent dose in the TRC cohort schizophrenia group (see Table S3C).

In the Stanley Medical Research Institute (SMRI) cohort, there were moderately strong, significant, inverse correlations of KATI mRNA expression with pH and PMI and between TDO mRNA and RIN in the schizophrenia group (see Table S3B). No other strong, significant correlations were obtained. See the Supplement Table S4 for sex differences in KP pathway mRNA expression and KP metabolites.

There was a trend toward a significant difference between the sexes (p=0.07) for KAT I in the TRC cohort. In the TRC cohort, smoking status did have a significant effect on TDO mRNA with those who smoked having elevated TDO mRNA levels, whereas no effect of smoking on TDO was detected in the SMRI cohort. Smoking status did not have any effect on KATI/II or KMO mRNAs (see Table S5).

No strong, significant correlations were detected between KP metabolites (KYN/TRP ratio, KYNA, KYNA/QUINA ratio) and duration of illness, lifetime or daily CPZ equivalent dose in the post-mortem cohort (Table S3 E).

**Kynurenine pathway enzymes and inflammatory status in TRC and SMRI postmortem cohorts, separated**

See Figure S1 for cytokine subgroup differences in KP mRNA expression. In the TRC cohort, we detected significant differences among cytokine subgroups in KAT II mRNA (F_(2,64)_=3.8, p=0.03, FDR p=0.12); while in the SMRI cohort there were significant differences in TDO (F_(2,54)_=3.5, p=0.04, FDR p=0.08) and KAT I mRNAs (F_(2,55)_=6.6, p<0.01, FDR p=0.04) with significantly higher enzyme mRNA in the high cytokine schizophrenia subgroup as compared to low cytokine schizophrenia and normal cytokine controls. As both cohorts showed activation of KAT enzyme activity in high cytokine schizophrenia, the cohorts were combined for further analyses to increase power in the final analysis. No subgroup differences were found in KMO mRNA (Supplemental Figure S1).

**Diagnostic differences of kynurenine pathway enzymes between patients with schizophrenia and controls**

Significantly higher KAT I mRNA expression (U=1784, p =0.04) and significantly higher TDO mRNA expression (t_130_=2.48, p=0.01) was found in schizophrenia patients when compared to controls. No significant diagnostic group differences where detected for KAT II or KMO expression (Figure S2).

**Demographics, cognition and regional brain volumes in patients versus controls -Living cohort**

Based on duration of illness and the PANSS scores, the patients were chronically ill and displayed mild to moderate symptom severity (Supplemental Table S2C). There were significant differences in age, education, and IQ between patients and controls (Supplemental Table S2c). See Supplemental Table S7 for a comparison of patients and controls in relation to cognitive domain scores and regional brain volumes. All cognitive domain scores (all p’s < 0.001) and prefrontal cortex volumes (Rostral middle frontal, p<0.001) were significantly reduced in schizophrenia relative to controls.

**Considerations of serotonin altering medication, sex and smoking on KP metabolites in schizophrenia and correlations of KP metabolites in patients and controls – Living cohort**

On the basis of a series of t-tests, there were no significant differences in TRP (p=0.11), KYN (p=0.19), KYNA (p=0.42), 3HK (p=0.29), or QUINA (p=0.13) levels when comparing those patients receiving serotonin-altering medications (SAM) (n=32) with those patients who were not receiving SAM (n=64). For details see Supplemental Table S8. See details on correlations of demographic and clinical variables with the KYN/TRP ratio in the schizophrenia and the healthy control groups in Supplemental Table S9. There was a mildly strong, significant relationship between KYN/TRP ratio with age in healthy controls and no other strong, significant correlations. KYN/TRP ratio did not correlate with mean daily CPZ equivalents dose (r=0.05, p=0.66) in patients. Smoking status (yes/no) did not have an influence on KP plasma metabolite levels in schizophrenia (Table S11). In patients with schizophrenia, current cigarette smoking status was not associated with inflammation group (low inflammation: smoking n=19/ non-smoking n=16; high inflammation: smoking n=13/ non-smoking n=17), chi-square test χ^2^=0.775, p=0.379.

A 2-way ANCOVA with diagnosis and sex as grouping factors, age as a covariate and Kyn/Trp ratio as the dependent variable revealed sex had no significant influence on Kyn/Trp ratio (F_1,172_=1.02, p=0.315).

**Schizophrenia cytokine subgroup differences on demographic, medication, symptom, cognitive, and brain volume measures – Living cohort**

Schizophrenia patients in the high cytokine subgroup had significantly greater premorbid intellectual estimates (p = 0.02) and significantly worse attention scores (p = 0.04) compared to low cytokine schizophrenia patients. No significant differences were found in current IQ, sex, age, education, PANSS scores, chlorpromazine equivalents, SAM, other cognitive domain scores or DLPFC volume between elevated and normal cytokine patient groups, see Supplemental Table S12.

**Supplemental Tables and Figures**

**Supplemental Table S1. Table indicating whether each statistical test met assumptions and covariate used in each analysis.**

| **Cohort** | **enzyme/metabolite** | **Homogeneity of variance** | **Group** | **Normal distribution** | **Covariate** |
| --- | --- | --- | --- | --- | --- |
|  | **enzyme** |  |  |  |  |
| **TRC** | KATI | **✓** | Con Low | **⌧** | **-** |
|  |  |  | Scz Low | **⌧** |  |
|  |  |  | Scz High | **✓** |  |
|  | KATII | **✓** | Con Low | **✓** | RIN |
|  |  |  | Scz Low | **✓** |  |
|  |  |  | Scz High | **⌧** |  |
|  | KMO | **✓** | Con Low | **✓** | **-** |
|  |  |  | Scz Low | **✓** |  |
|  |  |  | Scz High | **✓** |  |
|  | TDO | **✓** | Con Low | **✓** | RIN |
|  |  |  | Scz Low | **⌧** |  |
|  |  |  | Scz High | **✓** |  |
| **SMRI** | KATI | **✓** | Con Low | **⌧** | Age |
|  |  |  | Scz Low | **✓** |  |
|  |  |  | Scz High | **✓** |  |
|  | KATII | **✓** | Con Low | **✓** | RIN |
|  |  |  | Scz Low | **✓** |  |
|  |  |  | Scz High | **✓** |  |
|  | KMO | **✓** | Con Low | **✓** | **-** |
|  |  |  | Scz Low | **✓** |  |
|  |  |  | Scz High | **✓** |  |
|  | TDO | **✓** | Con Low | **✓** | **-** |
|  |  |  | Scz Low | **✓** |  |
|  |  |  | Scz High | **✓** |  |
| **Combined** | KATI | **✓** | Con Low | **✓** | PMI |
|  |  |  | Scz Low | **✓** |  |
|  |  |  | Scz High | **✓** |  |
|  | KATII | **✓** | Con Low | **✓** | **-** |
|  |  |  | Scz Low | **✓** |  |
|  |  |  | Scz High | **⌧** |  |
|  | KMO | **✓** | Con Low | **✓** | **-** |
|  |  |  | Scz Low | **✓** |  |
|  |  |  | Scz High | **✓** |  |
|  | TDO | **✓** | Con Low | **✓** | RIN |
|  |  |  | Scz Low | **⌧** |  |
|  |  |  | Scz High | **✓** |  |
| **Cohort** |  | **⬝** |  | **⬝** |  |
| **Post-mortem metabolites** | **metabolite** |  |  |  |  |
| **TRC** | Log KYN/TRP ratio | **✓** | Con Low | **✓** | PMI |
|  |  |  | Scz Low | **✓** |  |
|  |  |  | Scz High | **✓** |  |
|  | Log KYN | **✓** | Con Low | **✓** | PMI |
|  |  |  | Scz Low | **✓** |  |
|  |  |  | Scz High | **✓** |  |
|  | Log KYNA | **✓** | Con Low | **✓** | PMI |
|  |  |  | Scz Low | **⌧** |  |
|  |  |  | Scz High | **✓** |  |
|  | Log KYNA/QUINA ratio | **✓** | Con Low | **✓** | Age |
|  |  |  | Scz Low | **✓** |  |
|  |  |  | Scz High | **✓** |  |
| **Cohort** |  |  |  | **⬝** |  |
| **Living cohort metabolites** | **metabolite** |  |  |  |  |
| **Diagnostic differences** | TRP | **✓** | Con | **⌧** | Age |
|  |  |  | SZ | **✓** |  |
|  | KYN | **✓** | Con | **✓** |  |
|  |  |  | SZ | **✓** |  |
|  | Log KYNA | **✓** | Con | **✓** |  |
|  |  |  | SZ | **✓** |  |
|  | Log 3-HK | **✓** | Con | **✓** |  |
|  |  |  | SZ | **✓** |  |
|  | Log QUINA | **✓** | Con | **✓** |  |
|  |  |  | SZ | **✓** |  |
|  | Log KYN/TRP ratio | **✓** | Con | **✓** |  |
|  |  |  | SZ | **✓** |  |
| **Living cohort metabolites** | **metabolite** |  |  | **⬝** |  |
| **Inflammatory subroups** | Log KYN/TRP ratio | **✓** | Con Low | **✓** | Age |
|  |  |  | Con High | **✓** |  |
|  |  |  | Scz Low | **✓** |  |
|  |  |  | Scz High | **✓** |  |

TDO = Tryptophan-2,3-Dioxygenase, KMO = Kynurenine 3 Monooxygenase, KAT I/II = Kynurenine Amino Transferase I/II, TRP: tryptophan, KYN: kynurenine, KYNA: kynurenic acid, 3-HK: 3-hydroxykynurenine, QUINA: quinolinic acid, KYN/TRP Ratio, log = log transformed, Con = controls, low = low inflammatory subgroup, high = high inflammatory subgroup, Scz = schizophrenia, PMI = post-mortem interval, RIN = RNA integrity.

**✓**=Homogenity/Normality achieved, **** = Homogenity/Normality not achieved.

Postmortem cohort: For KYN/TRP ratio, KYN, KYNA/QUINA ratio, normality was achieved after log transformation. Covariates (age, RIN, PMI) were used in ANCOVA if the demographic factor correlated with the measurement of interest with one exception (tissue pH).

Living cohort: For KYNA, 3-HK, QUINA and the KYN/TRP ratio normality was achieved after log transformation. Age was included as covariate in the model because it is related to cognitive ability.

When a parametric distribution was not statistically achieved, additional visual inspection of the data was performed using histograms, boxplots, P-P plots and Q-Q plots. Thus, the data were visually inspected to confirm a relative bell-shaped distribution and the absence of outliers to enable use of parametric tests.

**Table S2**

**Demographics**

**a) Postmortem cohort 1 – New South Wales Tissue Resource Centre (TRC) cohort**

|  | **Patients**  **N=37** |  |  | **Controls**  **N=37** |  |  | **test value** | **df** | **p** |
| --- | --- | --- | --- | --- | --- | --- | --- | --- | --- |
|  | *Mean* | *sd* |  | *Mean* | *sd* |  |  |  |  |
| **Age** | 51.3 | 14.1 |  | 51.1 | 14.6 |  | t = 0.06 | 72 | 0.96 |
| **Sex** | 13 F/24 M |  |  | 7F/30M |  |  | χ^2^ = 2.47 | 1 | 0.12 |
| **Hemisphere** | 17R, 20 L |  |  | 23R, 14 L |  |  | χ^2^ = 1.96 | 1 | 0.16 |
| **pH** | 6.61 | 0.3 |  | 6.66 | 0.3 |  | t = 0.64 | 72 | 0.52 |
| **PMI (h)** | 28.8 | 14.1 |  | 24.8 | 11.0 |  | t = 1.26 | 72 | 0.21 |
| **RIN** | 7.3 | 0.6 |  | 7.3 | 0.6 |  | t = 0.24 | 72 | 0.81 |
| **Age of onset** | 23.7 | 0.1 |  | - |  |  |  |  |  |
| **DOI** | 27.6 | 2.3 |  | - |  |  |  |  |  |
| **CPZ** | 691.64 | 502.2 |  | - |  |  |  |  |  |
| **Manner of death** | 27 natural,  8 suicide,  1 accidental,  1 undetermined |  |  | 36 natural, 1 accidental |  |  |  |  |  |
| **Antipsychotics** | 30 FGAs,  6 SGAs,  1 both |  |  | - |  |  |  |  |  |
| **Antidepressant history** | 19 y, 18 n |  |  | - |  |  |  |  |  |
| **diagnosis** | 30 SZ/ 7 SA |  |  | - |  |  |  |  |  |

**b) Postmortem cohort 2 - Stanley Medical Research Institute (SMRI) cohort**

|  | **Patients**  **N=34** |  | | **Controls**  **N=35** |  |  | **test value** | **df** | **p** |
| --- | --- | --- | --- | --- | --- | --- | --- | --- | --- |
|  | *Mean* | | *sd* | *Mean* | *sd* |  |  |  |  |
| **Age** | 42.6 | | 8.5 | 43.8 | 7.4 |  | -.655 | 67 | 0.52 |
| **Sex** | 9F/26M | |  | 9F/25M |  |  | .943 | 1, 68 | 0.94 |
| **pH** | 6.5 | | .24 | 6.6 | 0.27 |  | -2.269 | 67 | 0.03* |
| **PMI (h)** | 31.4 | | 15.5 | 29.5 | 13.0 |  | .558 | 67 | 0.58 |
| **RIN** | 8.5 | | 0.6 | 8.3 | 0.7 |  | 1.153 | 67 | 0.25 |
| **Age of onset** | 21.3 | | 6.1 | - | - |  |  |  |  |
| **DOI** | 21.3 | | 10.1 | - | - |  |  |  |  |
| **AP Daily** | 10.71 | | 9.95 | - | - |  |  |  |  |
| **Manner of death** | 24 natural  7 suicide  4 accidental | |  | 35 natural |  |  |  |  |  |
| **Antidepressant history** | 9 y, 25 n | |  | 35 n |  |  |  |  |  |

**c) Living cohort**

|  | **Patients N=96** |  |  | **Controls N=81** |  |  | **test value** | **df** | **p value** |
| --- | --- | --- | --- | --- | --- | --- | --- | --- | --- |
|  | *Mean* | *sd* | *n* | *Mean* | *sd* | *n* |  |  |  |
| **Sex** | 59m/37f |  |  | 40m/41f |  |  | χ^2^ =2.1 | 1 | 0.15 |
| **Age** | 35.7 | 8.4 |  | 31.7 | 8.5 |  | t = 3.1 | 175 | 0.002* |
| **Education** | 12.5 | 2.4 |  | 14.6 | 2.2 |  | t = 6.0 | 175 | <0.001* |
| **WAIS-III IQ** | 90.9 | 13.2 |  | 107.1 | 14.8 |  | t =7.7 | 175 | <0.001* |
| **WTAR** | 102.2 | 9.1 |  | 107.7 | 8.8 |  | t =4.0 | 175 | <0.001* |
| **PANSS tot** | 60.3 | 16.5 |  | - | - |  |  |  | - |
| **PANSS pos** | 15.1 | 4.7 |  | - | - |  |  |  | - |
| **PANSS neg** | 14.5 | 6.2 |  | - | - |  |  |  | - |
| **Diagnosis** | 33SA/63SZ |  |  | - | - |  |  |  | - |
| **Age onset** | 22.8 | 5.6 |  | - | - |  |  |  | - |
| **Illness duration** | 12.9 | 7.6 |  | - | - |  |  |  | - |
| **CPZ** | 541.6 | 457.8 |  | - | - |  |  |  | - |
| **SAM no/yes** | 64/32 | - |  | - | - |  |  |  | - |

Notes. Means +/- standard deviations. PMI= postmortem interval, RIN= RNA integrity, DOI= duration of illness, M/F male/female. CPZ = mean daily chlorpromazine equivalent dose. FGAs= first generation antipsychotics, SGAs= second generation antipsychotics. AP daily = antipsychotic daily dose in fluphenazine equivalents, PANSS = Positive And Negative Syndrome Scale. WAIS-III = Wechsler Adult Intelligence Scale, 3^rd^ edition. WTAR = Wechsler Test of Adult Reading. SAM= Serotonin Altering Medications. SZ = Schizophrenia, SA = Schizoaffective Disorder, Age of illness onset, Illness duration, Education, Age in years, p-values refer to 2-sample t-tests or chi square tests comparing patients with controls. * significant at p<0.05.

**Table S3**

**Post mortem sample**

A) Kynurenine pathway correlations of metabolites with potentially confounding factors in healthy controls and schizophrenia – TRC cohort

|  |  |  | **RIN** | **pH** | **PMI** | **Age** |
| --- | --- | --- | --- | --- | --- | --- |
| **TDO** | HC | r | 0.25 | -0.09 | 0.16 | 0.18 |
|  |  | p | 0.15 | 0.61 | 0.36 | 0.31 |
|  |  | N | 35 | 35 | 35 | 35 |
|  | SZ | r | -0.26 | -0.22 | -0.07 | 0.03 |
|  |  | p | 0.13 | 0.21 | 0.69 | 0.88 |
|  |  | N | 34 | 34 | 34 | 34 |
| **KMO** | HC | r | 0.13 | 0.26 | 0.26 | -0.20 |
|  |  | p | 0.44 | 0.13 | 0.12 | 0.25 |
|  |  | N | 36 | 36 | 36 | 36 |
|  | SZ | r | 0.05 | 0.32 | 0.03 | -0.09 |
|  |  | p | 0.78 | 0.07 | 0.88 | 0.63 |
|  |  | N | 33 | 33 | 33 | 33 |
| **KAT I** | HC | r | -0.11 | -0.07 | -0.14 | 0.30 |
|  |  | p | 0.53 | 0.71 | 0.45 | 0.09 |
|  |  | N | 33 | 33 | 33 | 33 |
|  | SZ | r | -0.32 | -0.33 | 0.02 | 0.49**^**^** |
|  |  | P | 0.06 | 0.053 | 0.89 | 0.003 |
|  |  | N | 36 | 36 | 36 | 36 |
| **KAT II** | HC | r | -0.23 | -0.24 | -0.004 | -0.08 |
|  |  | p | 0.182 | 0.167 | 0.982 | 0.637 |
|  |  | N | 35 | 35 | 35 | 35 |
|  | SZ | r | -0.31 | -.45**^**^** | -0.05 | 0.23 |
|  |  | p | 0.07 | 0.006 | 0.79 | 0.18 |
|  |  | N | 36 | 36 | 36 | 36 |

B) Kynurenine pathway correlations of metabolites with potentially confounding factors in healthy controls and schizophrenia – SMRI cohort

|  |  |  | **RIN** | **pH** | **PMI** | **Age** |
| --- | --- | --- | --- | --- | --- | --- |
| **TDO** | HC | r | -.040 | -.227 | .029 | -.033 |
|  |  | p | .826 | .204 | .873 | .855 |
|  |  | N | 33 | 33 | 33 | 33 |
|  | SZ | r | -.567 | 0.062 | .046 | -.046 |
|  |  | p | .001** | .734 | .800 | .798 |
|  |  | N | 33 | 33 | 33 | 33 |
| **KMO** | HC | r | -.036 | .181 | .067 | .001 |
|  |  | p | .850 | .339 | .725 | .994 |
|  |  | N | 30 | 30 | 30 | 30 |
|  | SZ | r | -.319 | .098 | -.073 | -.238 |
|  |  | p | .092 | .614 | .707 | .213 |
|  |  | N | 29 | 29 | 29 | 29 |
| **KAT I** | HC | r | -.089 | -.130 | -.053 | -.020 |
|  |  | p | .629 | .477 | .773 | .912 |
|  |  | N | 31 | 32 | 32 | 32 |
|  | SZ | r | .047 | -.396 | -.452 | -.065 |
|  |  | p | .798 | .025* | .009** | .723 |
|  |  | N | 32 | 32 | 32 | 32 |
| **KAT II** | HC | r | -.027 | -.011 | -.279 | .189 |
|  |  | p | .883 | .954 | .122 | .301 |
|  |  | N | 32 | 32 | 32 | 32 |
|  | SZ | r | -.080 | .043 | -.061 | -.098 |
|  |  | p | .663 | .814 | .739 | .593 |
|  |  | N | 32 | 32 | 32 | 32 |

C) Kynurenine pathway mRNA expression correlations with mean daily CPZ dose and illness duration in schizophrenia – TRC cohort

|  |  | **DOI** | **Lifetime CPZ** | **Daily CPZ** |
| --- | --- | --- | --- | --- |
| **TDO** | r | 0.05 | 0.03 | 0.003 |
|  | p | 0.79 | 0.85 | 0.99 |
|  | N | 34 | 34 | 34 |
| **KMO** | r | -0.13 | -0.33 | -.44**^**^** |
|  | p | 0.48 | 0.06 | 0.01 |
|  | N | 33 | 33 | 33 |
| **KAT I** | r | 0.42**^*^** | 0.46**^**^** | 0.40**^*^** |
|  | p | 0.01 | 0.005 | 0.017 |
|  | N | 36 | 36 | 36 |
| **KAT II** | r | 0.15 | 0.21 | 0.21 |
|  | p | 0.37 | 0.23 | 0.22 |
|  | N | 36 | 36 | 36 |

D) Kynurenine pathway mRNA expression correlations with mean daily Fluphenazine dose and illness duration (DOI) in schizophrenia – SMRI cohort

|  |  |  | **DOI** | **Lifetime AP**  **Fluphenazine equiv.** |
| --- | --- | --- | --- | --- |
| **TDO** | SZ | r | .06 | .20 |
|  |  | p | .74 | .26 |
|  |  | N | 33 | 33 |
| **KMO** | SZ | r | -.231 | -.170 |
|  |  | p | .23 | .38 |
|  |  | N | 29 | 29 |
| **KAT I** | SZ | r | .06 | .07 |
|  |  | p | .74 | .70 |
|  |  | N | 32 | 32 |
| **KAT II** | SZ | r | -.01 | .19 |
|  |  | p | .95 | .30 |
|  |  | N | 32 | 32 |

E) Kynurenine metabolite correlations with mean daily CPZ dose and illness duration in schizophrenia – TRC cohort

|  |  | **DOI** | **Lifetime CPZ** | **Daily CPZ** |
| --- | --- | --- | --- | --- |
| **KYN/TRP ratio** | r | 0.12 | 0.29 | 0.31 |
|  | p | 0.49 | 0.10 | 0.08 |
|  | N | 34 | 34 | 34 |
| **KYNA** | r | 0.05 | -0.05 | 0.01 |
|  | p | 0.77 | 0.79 | 0.96 |
|  | N | 34 | 34 | 34 |
| **KYNA/QUINA ratio** | r | -0.28 | -0.22 | -0.12 |
|  | p | 0.116 | 0.21 | 0.51 |
|  | N | 34 | 34 | 34 |

Notes. TDO = Tryptophan-2,3-Dioxygenase, KMO = Kynurenine 3 Monooxygenase, KATI/II = Kynurenine Amino Transferase I/II, PMI= postmortem interval, RIN= RNA integrity, KYN/TRP ratio = Kynurenine/Tryptophan ratio, KYNA = Kynurenic Acid, KYNA/QUINA ratio = Kynurenic Acid/Quinolinic Acid ratio, DOI= duration of illness, CPZ = mean daily chlorpromazine equivalent dose. SZ = Schizophrenia, HC = Healthy controls, Age in years, p-values refer to Pearson or Spearman correlations. **^*^** significant at p<0.05, ** significant at p<0.01.

**Table S4**

**Sex differences in Kynurenine pathway mRNA expression and metabolites in the postmortem sample**

A) TRC cohort

|  |  | **Mean** | **t** | **df** | **p** |
| --- | --- | --- | --- | --- | --- |
| **TDO** | Male | 3.66 | -.332 | 64 | .741 |
|  | Female | 3.81 |  |  |  |
| **KMO** | Male | 15.11 | 1.391 | 63 | .169 |
|  | Female | 12.63 |  |  |  |
| **KAT I** | Male | 4.32 | -1.830 | 64 | .072 |
|  | Female | 4.71 |  |  |  |
| **KAT II** | Male | 4.17 | -1.295 | 66 | .200 |
|  | Female | 4.47 |  |  |  |

B) SMRI cohort

|  |  | **Mean** | **t** | **df** | **p** |
| --- | --- | --- | --- | --- | --- |
| **TDO** | Male | 3.91 | .589 | 64 | 0.56 |
|  | Female | 3.54 |  |  |  |
| **KMO** | Male | 13.78 | .605 | 58 | .55 |
|  | Female | 12.53 |  |  |  |
| **KAT I** | Male | 4.81 | .122 | 66 | .90 |
|  | Female | 4.76 |  |  |  |
| **KAT II** | Male | 4.33 | 1.047 | 67 | .299 |
|  | Female | 3.96 |  |  |  |

C) TRC cohort - metabolites

|  |  | **Mean** | **t** | **df** | **p** |
| --- | --- | --- | --- | --- | --- |
| **TRP** | Male | 16.4 | -0.57 | 73 | 0.57 |
|  | Female | 17.5 |  |  |  |
| **KYN** | Male | 0.38 | -0.97 | 73 | 0.33 |
|  | Female | 0.39 |  |  |  |
| **KYNA** | Male | 89.7 | -1.34 | 73 | 0.19 |
|  | Female | 94.0 |  |  |  |
| **QUINA** | Male | 51.0 | -1.28 | 73 | 0.20 |
|  | Female | 59.8 |  |  |  |

TDO = Tryptophan-2,3-Dioxygenase, KMO = Kynurenine 3 Monooxygenase, KATI/II = Kynurenine Amino Transferase I/II. TRP = Tryptophan, KYN = Kynurenine, KYNA = Kynurenic Acid, QUINA = Quinolinic Acid. p-values refer to 2-sample t-tests.

**Table S5**

**Smoking status and KP enzyme mRNA in the postmortem cohorts**

A) TRC cohort (15 with unknown smoking status)

|  | **smoking status** | **n** | **mean** | **sd** | **t test** | **p** |
| --- | --- | --- | --- | --- | --- | --- |
| **KAT I** | no | 23 | 4.26 | .86 | 1.11 | 0.27 |
|  | yes | 31 | 4.51 | .76 |  |  |
| **KAT II** | no | 26 | 4.21 | .91 | 0.34 | 0.74 |
|  | yes | 30 | 4.28 | .73 |  |  |
| **KMO** | no | 26 | 15.11 | 6.29 | -.25 | 0.81 |
|  | yes | 30 | 14.66 | 7.28 |  |  |
| **TDO** | no | 25 | 3.08 | 1.19 | 2.69 | 0.01* |
|  | yes | 30 | 4.15 | 1.74 |  |  |

B) SMRI cohort (24 with unknown smoking status)

|  | **smoking status** | **n** | **mean** | **sd** | **t test** | **p** |
| --- | --- | --- | --- | --- | --- | --- |
| **KAT I** | no | 31 | 4.91 | 1.75 | .30 | 0.77 |
|  | yes | 13 | 5.05 | 1.23 |  |  |
| **KAT II** | no | 32 | 4.58 | 1.45 | 1.18 | 0.25 |
|  | yes | 13 | 4.06 | 1.29 |  |  |
| **KMO** | no | 27 | 13.59 | 7.62 | .71 | 0.49 |
|  | yes | 11 | 11.95 | 6.03 |  |  |
| **TDO** | no | 30 | 4.23 | 2.79 | .67 | 0.51 |
|  | yes | 13 | 3.65 | 2.51 |  |  |

C) TRC cohort - metabolites (20 with unknown smoking status)

|  | **smoking status** | **n** | **mean** | **sd** | **t test** | **p** |
| --- | --- | --- | --- | --- | --- | --- |
| **KYN/TRP ratio** | no | 26 | 2.30 | .35 | 1.06 | 0.29 |
|  | yes | 29 | 2.21 | .23 |  |  |
| **KYNA** | no | 26 | 2.76 | .47 | 0.95 | 0.35 |
|  | yes | 29 | 2.67 | .25 |  |  |
| **KYNA/QUINA ratio** | no | 24 | .093 | .04 | 0.16 | 0.87 |
|  | yes | 29 | .076 | .04 |  |  |

Notes. TDO = Tryptophan-2,3-Dioxygenase, KMO = Kynurenine 3 Monooxygenase, KATI/II = Kynurenine Amino Transferase I/II. KYN/TRP ratio = Kynurenine/Tryptophan ratio, KYNA = Kynurenic Acid, KYNA/QUINA ratio = Kynurenic Acid/Quinolinic Acid ratio. p-values refer to 2-sample t-tests. * significant at p<0.05.

**Table S6**

**A) Correlations between KP enzyme and proinflammatory cytokine mRNA in schizophrenia patients – TRC cohort**

|  | | **TDO** | **KAT I** | **KAT II** | **KMO** |
| --- | --- | --- | --- | --- | --- |
| **IL1-β** | ρ | .199 | .510^**^ | .281 | -.469^**^ |
|  | p | .274 | .002 | .108 | .008 |
|  | n | 32 | 34 | 34 | 31 |
| **IL-6** | ρ | .179 | .442^**^ | .418^*^ | -.188 |
|  | p | .318 | .009 | .014 | .311 |
|  | N | 33 | 34 | 34 | 31 |
| **IL-8** | ρ | .137 | .125 | .431^*^ | .218 |
|  | p | .486 | .511 | .017 | .275 |
|  | n | 28 | 30 | 30 | 27 |
| **SERPINA3** | ρ | .188 | .538^**^ | .509^**^ | -.555^**^ |
|  | p | .321 | .001 | .003 | .002 |
|  | n | 30 | 32 | 32 | 29 |

**B) Correlations between KP enzyme and inflammatory marker mRNAs in schizophrenia patients – SMRI cohort**

|  | | **TDO** | **KAT I** | **KAT II** | **KMO** |
| --- | --- | --- | --- | --- | --- |
| **IL1-β** | ρ | .177 | .366 | .076 | .047 |
|  | p | .377 | .056 | .702 | .824 |
|  | n | 27 | 28 | 28 | 25 |
| **IL-6** | ρ | .246 | .358* | -.046 | .181 |
|  | p | .190 | .048 | .808 | .365 |
|  | N | 30 | 31 | 31 | 27 |
| **IL-8** | ρ | .077 | -.069 | -.070 | -.231 |
|  | p | .719 | .750 | .747 | .288 |
|  | n | 24 | 24 | 24 | 23 |
| **SERPINA3** | ρ | .263 | .405* | .257 | .019 |
|  | p | .152 | .022 | .155 | .925 |
|  | n | 31 | 32 | 32 | 28 |

TDO = Tryptophan-2,3-Dioxygenase, KMO = Kynurenine 3 Monooxygenase, KATI/II = Kynurenine Amino Transferase I/II. p-values refer to Spearman rank correlations. * significant at p<0.05.

**Table S7**

**Cognitive domain z-scores and prefrontal cortex volumes in patients with schizophrenia and healthy controls - Living cohort**

|  | **schizophrenia** |  |  | **healthy controls** |  |  | **F-value** | **p value** |
| --- | --- | --- | --- | --- | --- | --- | --- | --- |
|  | *Mean* | *sd* | *n* | *Mean* | *sd* | *n* |  |  |
| **Verbal memory** | -2.40 | 2.4 | 96 | 0.29 | 2.1 | 81 | 38.4 | <0.001* |
| **Language** | -1.08 | 1.6 | 96 | -0.11 | 1.7 | 81 | 9.3 | <0.001* |
| **Working memory** | -2.10 | 1.6 | 96 | -0.25 | 1.6 | 81 | 32.7 | <0.001* |
| **Processing Speed** | -1.40 | 0.8 | 96 | 0.03 | 1.0 | 81 | 57.3 | <0.001* |
| **Perceptual Organization** | -0.82 | 0.9 | 96 | -0.003 | 1.0 | 81 | 15.2 | <0.001* |
| **Attention** | -1.53 | 1.5 | 72 | -0.01 | 1.0 | 64 | 25.4 | <0.001* |
| **Rostral Middle Frontal gyrus** | 32375.2 | 4434.6 | 60 | 34190.5 | 4407.6 | 61 | 37.4 | <0.001* |

Means +/- standard deviations. N is sample size. Cognitive domain scores converted to Z-scores. Rostral middle frontal volume in µL, Cognitive domains of verbal memory, language, working memory, processing speed, perceptual organization and attention were formed using previously reported methods.^14^ p-values refer to ANCOVAs comparing patients with controls for cognitive measures, covaried for age, and an ANCOVA for volumetric measures, covaried for age and ICV. * significant at p<0.05.

**Table S8**

**Correlations of demographic and clinical variables with KYN/TRP Ratio in Patients with Schizophrenia and Healthy Controls – Living cohort**

|  | **healthy control**  **(N=81, r, p)** | **schizophrenia total**  **(N=96, r, p)** |
| --- | --- | --- |
| **Age** | r=0.25 p=0.03* | r=-0.16 p=0.12 |
| **Education** | r=0.00 p=0.99 | r=0.01 p=0.89 |
| **Age of onset** | - | N=96 r=-0.05 p=0.65 |
| **Illness duration** | - | N=96 r=-0.15 p=0.16 |
| **Chlorpromazine equivalents** | - | N=96 r=0.05 p=0.66 |
| **Imipramine equivalents** | - | N=96 r=0.17 p=0.10 |

Notes. Correlation of demographic and clinical variables with Kynurenine/Tryptophan (KYN/TRP) ratio in patients with schizophrenia or healthy controls and sex differences among groups for the total sample at p<0.05. Age, Age of onset, Illness duration in years. Mean daily dose of antipsychotic medication was converted to chlorpromazine equivalents^1^ and antidepressant medication was converted to approximate daily mean imipramine equivalents dose according to **^15, 16^**. * significant at p<0.05.

**Table S9**

**Serotonin Altering Medication (SAM) and plasma Kynurenine Pathway metabolites – Living cohort**

|  | **schizophrenia no SAM**  **N = 64** | | **schizophrenia with SAM**  **N = 32** | | **T-value** | **df** |  | **p** |
| --- | --- | --- | --- | --- | --- | --- | --- | --- |
|  | **mean** | **sd** | **mean** | **sd** |  |  |  |  |
| **TRP (μM)** | 43.7 | 8.3 | 40.9 | 6.8 | 1.6 | 94 |  | 0.11 |
| **KYN (μM)** | 1.6 | 0.3 | 1.8 | 0.5 | 1.3 | 94 |  | 0.19 |
| **KYNA (nM)** | 38.9 | 13.1 | 41.2 | 11.8 | 0.8 | 94 |  | 0.42 |
| **3-HK (nM)** | 38.1 | 15.2 | 41.7 | 15.2 | 1.1 | 94 |  | 0.29 |
| **QUINA (nM)** | 262.7 | 74.4 | 290.8 | 97.1 | 1.5 | 92 |  | 0.13 |

SAM: Serotonin Altering Medication, TRP: tryptophan, KYN: kynurenine, KYNA: kynurenic acid, 3-HK: 3-hydroxykynurenine, QUINA: quinolinic acid, p-values refer to two sample t-tests.

**Table S10**

**Influence of smoking status on KP plasma metabolites in patients with schizophrenia – Living cohort**

|  | **smoking status** | **n** | **mean** | **sd** | **t test** | **p** |
| --- | --- | --- | --- | --- | --- | --- |
| **KYN/TRP Ratio** | no | 37 | 43.49 | 12.14 | 1.57 | 0.12 |
|  | yes | 38 | 39.76 | 8.08 |  |  |
| **TRP** | no | 35 | 42.26 | 8.21 | -0.12 | 0.90 |
|  | yes | 35 | 42.49 | 7.65 |  |  |
| **KYN** | no | 37 | 1.71 | 0.42 | 0.33 | 0.74 |
|  | yes | 36 | 1.68 | 0.39 |  |  |
| **KYNA** | no | 37 | 41.65 | 13.21 | 1.28 | 0.21 |
|  | yes | 36 | 37.76 | 12.81 |  |  |
| **3HK** | no | 37 | 39.37 | 16.27 | 0.11 | 0.92 |
|  | yes | 37 | 38.99 | 14.58 |  |  |
| **QUINA** | no | 33 | 276.21 | 86.78 | 0.98 | 0.33 |
|  | yes | 37 | 258.38 | 65.01 |  |  |

TRP: tryptophan, KYN: kynurenine, KYNA: kynurenic acid, 3-HK: 3-hydroxykynurenine, QUINA: quinolinic acid, KYN/TRP Ratio: kynurenine to tryptophan ratio, p-values refer to two sample t-tests.

**Table S11**

**Linear regression model between attention performance, Kyn/Trp ratio and PANSS scores.**

| Included Variable |  | Beta | t | sign | correl |
| --- | --- | --- | --- | --- | --- |
|  | Panss neg | -0.43 | -2.5 | 0.021 |  |
|  | Kyn/Trp ratio | -0.38 | -2.2 | 0.038 |  |
| Excluded Variable |  |  |  |  |  |
|  | Panss pos | 0.21 | 1.1 | 0.30 | 0.23 |
|  | Panss gen | -0.22 | -0.96 | 0.35 | -0.21 |

A stepwise linear regression was performed between attention and the following variables: Kyn/Trp ratio, PANSS pos, PANNS neg and PANNS gen scores (F(1,23)=6.5, p=0.06). In addition to Kyn/Trp ratio, only PANSS negative score was significantly associated with attention performance.

**Table S12**

**Demographic and Clinical Characteristics, cognitive abilities, and prefrontal cortex volumes of Schizophrenia Patients in low and high cytokine subgroups – Living cohort**

|  | **SZ low cytokines** |  |  | **SZ high cytokines** |  |  | **Test value** | **df** | **p-value** |
| --- | --- | --- | --- | --- | --- | --- | --- | --- | --- |
|  | **mean** | **sd** | **n** | **mean** | **sd** | **n** |  |  |  |
| **sex** | 18m/24f |  |  | 16m/23f |  |  | χ^2^ = 0.03 | 1 | 0.87 |
| **age** | 35.2 | 9.9 | 42 | 36.5 | 7.2 | 39 | t = -0.7 | 79 | 0.51 |
| **Education** | 12.5 | 2.3 | 42 | 12.7 | 2.3 | 39 | t = -0.4 | 79 | 0.67 |
| **WAIS-III IQ** | 90.7 | 13.0 | 42 | 91.8 | 13.3 | 39 | t = -0.4 | 79 | 0.71 |
| **WTAR** | 100.9 | 9.4 | 42 | 105.3 | 7.6 | 39 | t = -2.3 | 79 | 0.02* |
| **PANSS total** | 60.7 | 15.2 | 42 | 59.6 | 17.7 | 39 | t = 0.3 | 79 | 0.76 |
| **PANSS positive** | 15.2 | 4.4 | 42 | 15.2 | 4.4 | 39 | t = -0.04 | 79 | 0.97 |
| **PANSS negative** | 15.0 | 5.7 | 42 | 13.7 | 6.6 | 39 | t = 0.9 | 79 | 0.36 |
| **Age of onset** | 22.4 | 6.6 | 42 | 22.8 | 4.6 | 39 | t = -0.3 | 79 | 0.77 |
| **DOI** | 12.6 | 7.8 | 42 | 13.8 | 7.4 | 39 | t = -0.7 | 79 | 0.48 |
| **CPZ** | 523.2 | 471.6 | 42 | 569.0 | 500.8 | 39 | t = -0.4 | 79 | 0.67 |
| **SAM** | 24n/18y |  |  | 23n/14y |  |  | χ^2^ =0.2 | 1 | 0.65 |
| **Verbal memory** | -2.0 | 1.9 | 42 | -2.6 | 2.2 | 39 | t = 1.3 | 79 | 0.21 |
| **Language** | -1.0 | 1.8 | 42 | -1.0 | 1.6 | 39 | t = -0.1 | 79 | 0.91 |
| **Working memory** | -2.2 | 1.5 | 42 | -2.0 | 1.8 | 39 | t = -0.5 | 79 | 0.63 |
| **Processing Speed** | -1.4 | 0.8 | 42 | -1.4 | 0.7 | 39 | t = 0.4 | 79 | 0.72 |
| **Perceptual Organisation** | -0.86 | 0.9 | 42 | -0.7 | 0.9 | 39 | t = -0.9 | 79 | 0.38 |
| **Attention** | -1.17 | 1.4 | 35 | -2.0 | 1.7 | 24 | t = -2.1 | 57 | 0.04* |
| **Rostral middle frontal volume** | 32494.5 | 4808.6 | 27 | 31851.3 | 3149.4 | 23 | t = 0.55 | 48 | 0.59 |

Means +/- standard deviations. M/F male/female. CPZ = mean daily chlorpromazine equivalent dose. PANSS = Positive And Negative Syndrome Scale. WAIS-III IQ = Wechsler Adult Intelligence Scale, 3^rd^ edition. WTAR = Wechsler Test of Adult Reading. SAM= Serotonin Altering Medications. DOI= duration of illness, Age of onset = Age of illness onset, Education, Age in years. Cognitive domain scores converted to Z-scores. Rostral middle frontal volume in µL, Cognitive domains of verbal memory, language, working memory, processing speed, perceptual organization and attention were formed using previously reported methods. p-values refer to 2-sample t-tests or chi square tests comparing patients with controls. * significant at p<0.05.

**Table S13**

**A) Correlations between plasma KYN/TRP ratio and proinflammatory markers in schizophrenia patients – Living cohort**

|  | **Plasma KYN/TRP ratio, cytokine mRNAs and CRP in schizophrenia patients** | | |
| --- | --- | --- | --- |
|  | **R** | **p** | **n** |
| **IL-1β** | 0.23* | 0.033 | 88 |
| **IL-6** | 0.17 | 0.11 | 88 |
| **IL-8** | 0.48 | 0.66 | 88 |
| **IL-18** | 0.17 | 0.10 | 88 |
| **CRP** | 0.28* | 0.010 | 84 |

**B) Correlations between plasma KYN/TRP ratio and proinflammatory markers in healthy controls – Living cohort**

|  | **Plasma KYN/TRP ratio, cytokine mRNAs and CRP in controls** | | |
| --- | --- | --- | --- |
|  | **R** | **p** | **n** |
| **IL-1β** | -0.14 | 0.26 | 71 |
| **IL-6** | -0.22 | 0.07 | 71 |
| **IL-8** | -0.14 | 0.26 | 71 |
| **IL-18** | -0.17 | 0.16 | 71 |
| **CRP** | 0.13 | 0.28 | 67 |

IL = Interleukin mRNA, CRP = C reactive protein, R = Pearson Correlation, p-value, n = number, * significant at p<0.05.

**Figure S1**
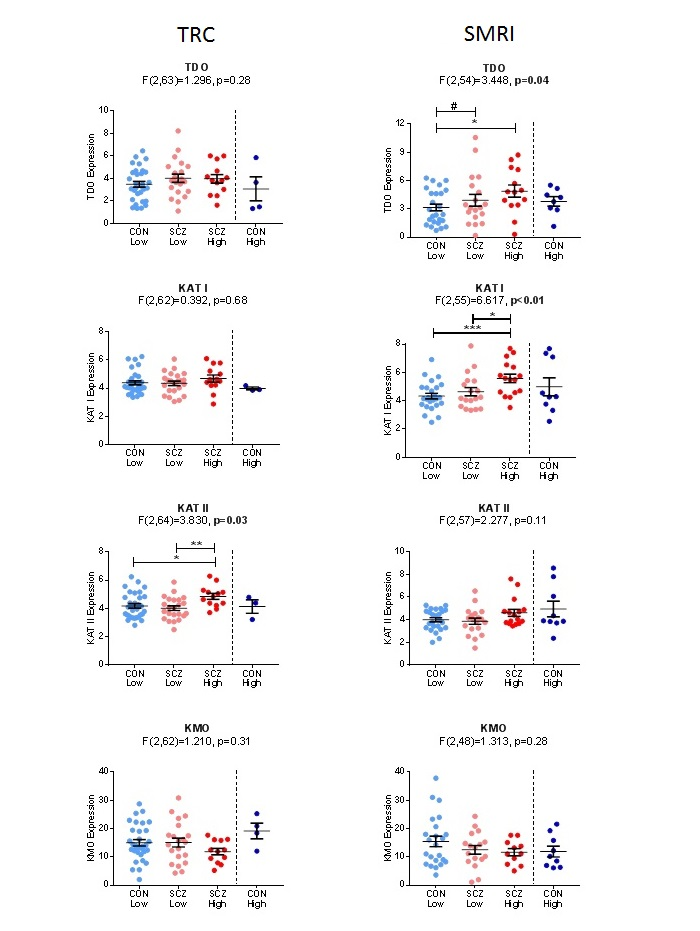


Cytokine subgroup differences of Kynurenine Pathway enzyme mRNA expression in postmortem brain tissue. Tryptophan 2,3-dioxygenase (TDO) mRNA, Kynurenine Aminotransferases I (KAT I) and II (KAT II) and Kynurnenine 3- Monooxygenase (KMO) mRNA expression among cytokine subgroups, separated for the TRC and the SMRI postmortem cohorts. # trend at p<0.1, * significant at p<0.05, ** significant at p<0.01, *** significant at p<0.001.

**Figure S2**

Diagnostic differences (CON = healthy controls, SCZ = schizophrenia) of kynurenine pathway enzyme mRNA expression in postmortem brain tissue. Kynurenine Aminotransferases I (KAT I) and II (KAT II), Kynurnenine 3- Monooxygenase (KMO) and Tryptophan 2,3-dioxygenase (TDO) mRNA of combined cohorts (TRC+SMRI). KAT I was non-parametric so the error bars are median +/- 95% CI. KAT II was covaried for RIN. * significant at p<0.05.

**Figure S3**

**
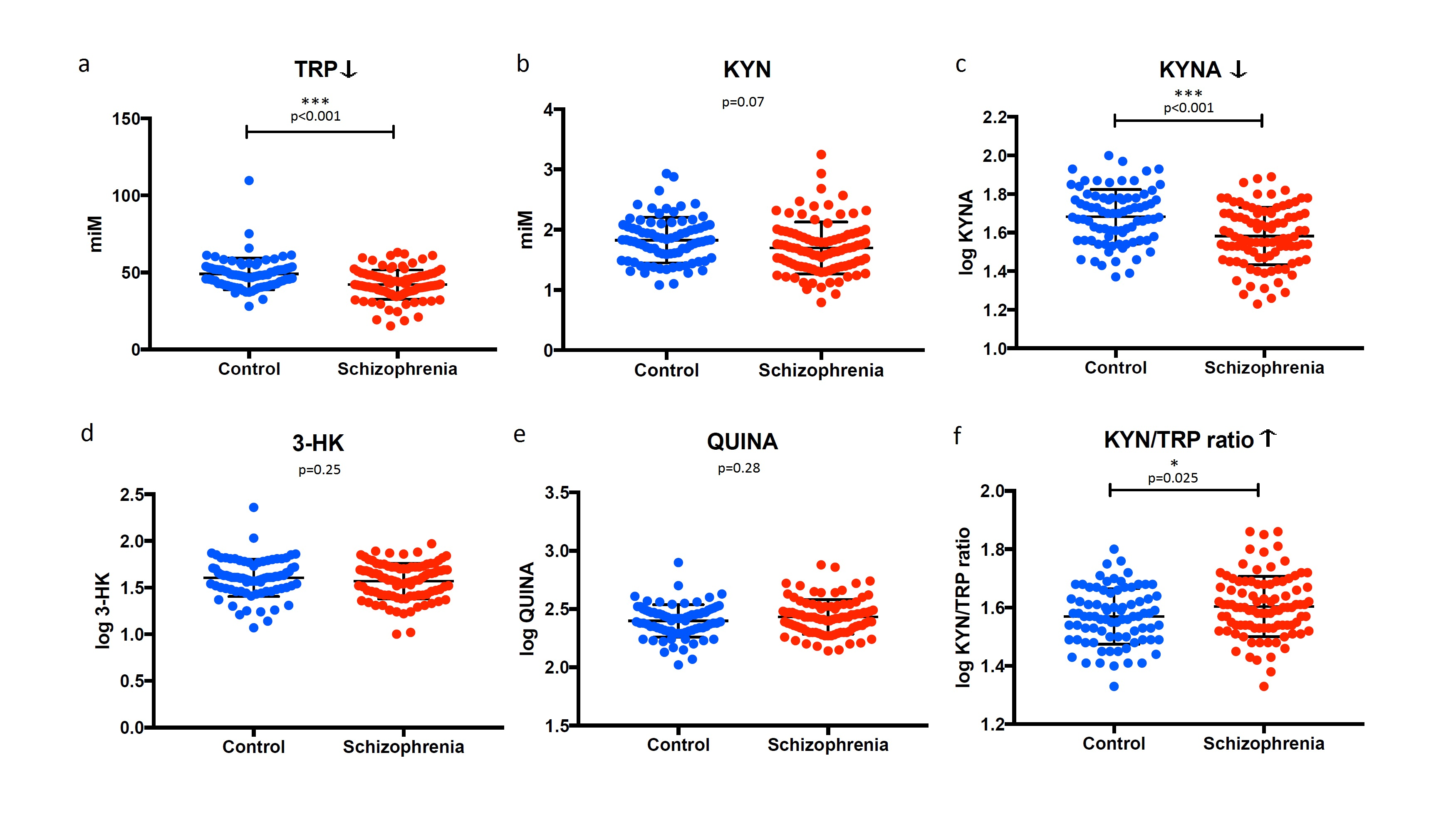
**

Comparison of Kynurenine Plasma Metabolites based on diagnostic groups. a) TRP Tryptophan (μM), b) KYN Kynurenine (μM), c) KYNA Kynurenic Acid (nM), d) 3-HK 3-Hydroxykynurenine (nM), e) QUINA Quinolinic Acid (nM), f) KYN/TRP Ratio Kynurenine/ Tryptophan ratio, p-values refer to F-tests comparing patients with controls. *significant at p≤0.05, FDR corrected, two-tailed.

**References**

1. Fillman SG, Cloonan N, Catts VS, Miller LC, Wong J, McCrossin T *et al.* Increased inflammatory markers identified in the dorsolateral prefrontal cortex of individuals with schizophrenia. *Mol Psychiatry* 2013; **18**(2)**:** 206-214.

2. Fillman SG, Sinclair D, Fung SJ, Webster MJ, Shannon Weickert C. Markers of inflammation and stress distinguish subsets of individuals with schizophrenia and bipolar disorder. *Transl Psychiatry* 2014; **4:** e365.

3. Boerrigter D, Weickert TW, Lenroot R, O'Donnell M, Galletly C, Liu D *et al.* Using blood cytokine measures to define high inflammatory biotype of schizophrenia and schizoaffective disorder. *J Neuroinflammation* 2017; **14**(1)**:** 188.

4. Leucht S, Wahlbeck K, Hamann J, Kissling W. New generation antipsychotics versus low-potency conventional antipsychotics: a systematic review and meta-analysis. *Lancet* 2003; **361**(9369)**:** 1581-1589.

5. Kay SR, Fiszbein A, Opler LA. The positive and negative syndrome scale (PANSS) for schizophrenia. *Schizophr Bull* 1987; **13**(2)**:** 261-276.

6. Lim CK, Bilgin A, Lovejoy DB, Tan V, Bustamante S, Taylor BV *et al.* Kynurenine pathway metabolomics predicts and provides mechanistic insight into multiple sclerosis progression. *Sci Rep* 2017; **7:** 41473.

7. Smythe GA, Braga O, Brew BJ, Grant RS, Guillemin GJ, Kerr SJ *et al.* Concurrent quantification of quinolinic, picolinic, and nicotinic acids using electron-capture negative-ion gas chromatography-mass spectrometry. *Anal Biochem* 2002; **301**(1)**:** 21-26.

8. Jacomb I, Stanton C, Vasudevan R, Powell H, O'Donnell M, Lenroot R *et al.* C-Reactive Protein: Higher During Acute Psychotic Episodes and Related to Cortical Thickness in Schizophrenia and Healthy Controls. *Front Immunol* 2018; **9:** 2230.

9. Wechsler D. Wechsler Adult Intelligence Scale. , The Psychological Corporation: San Antonio, Texas. 1997.

10. Wechsler D. Wechsler Test of Adult Reading. , The Psychological Corporation: San Antonio, Texas. 2001.

11. Wechsler D. *Wechsler Memory Scale-Revised*. Psychological Corporation: San Antonio, 1987.

12. Lezak M. *Neuropsychological Assessment*. 4th edition edn. Oxford University Press, New York2004.

13. Reitan R. *The Halstead-Reitan Neuropsychological Test Battery: Therapy and Clinical Interpretation*: Tucson, AZ, USA, 1985.

14. Moore L, Kyaw M, Vercammen A, Lenroot R, Kulkarni J, Curtis J *et al.* Serum testosterone levels are related to cognitive function in men with schizophrenia. *Psychoneuroendocrinology* 2013; **38**(9)**:** 1717-1728.

15. Baldessarini RJ, Tondo L, Ghiani C, Lepri B. Illness risk following rapid versus gradual discontinuation of antidepressants. *Am J Psychiatry* 2010; **167**(8)**:** 934-941.

16. Hayasaka Y, Purgato M, Magni LR, Ogawa Y, Takeshima N, Cipriani A *et al.* Dose equivalents of antidepressants: Evidence-based recommendations from randomized controlled trials. *J Affect Disord* 2015; **180:** 179-184.
